# SPOCK1 is a novel inducer of epithelial to mesenchymal transition in drug-induced gingival overgrowth

**DOI:** 10.1038/s41598-020-66660-z

**Published:** 2020-06-17

**Authors:** Rehab Alshargabi, Tomomi Sano, Akiko Yamashita, Aiko Takano, Taiki Sanada, Misaki Iwashita, Takanori Shinjo, Takao Fukuda, Terukazu Sanui, Shosei Kishida, Fusanori Nishimura

**Affiliations:** 1 Section of Periodontology, Division of Oral Rehabilitation, Kyushu University Faculty of Dental Science, Fukuoka, Japan, 3-1-1 Maidashi, Higashi-ku, Fukuoka, 812-8582 Japan; 2 Department of Biochemistry and Genetics, Kagoshima University Graduate school of Dental and Medical Sciences, Kagoshima, Japan, 8-35-1 Sakuragaoka, Kagoshima, 890-8544 Japan

**Keywords:** Cell biology, Diseases, Pathogenesis

## Abstract

Few studies have investigated the role of extracellular-matrix proteoglycans in the pathogenesis of drug-induced gingival overgrowth (DIGO). SPOCK1 is an extracellular proteoglycan that induces epithelial to mesenchymal transition (EMT) in several cancer cell lines and exhibits protease-inhibitory activity. However, the role of SPOCK1 in non-cancerous diseases such as DIGO has not been well-addressed. We demonstrated that the expression of SPOCK1, TGF-β1, and MMP-9 in calcium channel blocker-induced gingival overgrowth is higher than that in non-overgrowth tissues. Transgenic mice overexpressing *Spock*1 developed obvious gingival-overgrowth and fibrosis phenotypes, and positively correlated with EMT-like changes. Furthermore, *in vitro* data indicated a tri-directional interaction between SPOCK1, TGF-β1, and MMP-9 that led to gingival overgrowth. Our study shows that SPOCK1 up-regulation in a noncancerous disease and SPOCK1-induced EMT in gingival overgrowth occurs via cooperation and crosstalk between several potential signaling pathways. Therefore, SPOCK1 is a novel therapeutic target for gingival overgrowth and its expression is a potential risk of EMT induction in cancerous lesions.

## Introduction

Drug-induced gingival overgrowth (DIGO) refers to bead-like enlargements of the interdental papillae that extend to the facial and lingual margins^1^. DIGO is a side effect of some drugs such as anticonvulsants (phenytoin, PHE), immunosuppressants (cyclosporine A, CsA), and calcium channel blockers (nifedipine, NFD)^1^. Despite pharmacological diversity among these causative drugs, they are suggested to share similar mechanisms at the cellular level^2^. A number of cellular and molecular mechanisms in epithelial and connective tissues may be involved in the pathogenesis of DIGO. For example, *in vitro* studies revealed phenotypic changes in both keratinocytes and fibroblasts^3^ and at the histopathological level, all forms of DIGO share similar features, including parakeratinized squamous epithelium with elongated rete pegs that extend deep into the connective tissue, and collagen accumulation within lamina propria^4,5^

Along with collagen accumulation, non-collagenous components of the extra-cellular matrix (ECM) like glycoaminoglycans (GAG) and proteoglycans (PGs) are reported to be increased with PHE, CsA, and NFD treatment^6–12^. The accumulating ECM may occur due to an imbalance between ECM synthesis and degradation in instances where these drugs are indicated^13^. ECM degradation mainly occurs through the activity of matrix metalloproteinases (MMPs) or cathepsins. Cathepsins are lysosomal enzymes that are responsible for the intracellular breakdown of up to 90% of long-lived cellular proteins^14^. Interestingly, a previous study reported that mice deficient in the *cathepsin L* gene manifested gingival overgrowth^13^. Conversely, SPOCK1, which was previously known as testican-1, is an extracellular proteoglycan that belongs to the secreted protein acidic and rich in cysteine (SPARC) family with a unique multi-domain protein core and glycosaminoglycan side chain that has different biological functions. SPOCK1 is composed of five domains, including three domains that have homology to three different classes of protease inhibitors which relate to its specific inhibitory function of cathepsin L activity^15,16^.

Gingival overgrowth is characterized by a thickening of the epithelium and elongated rete pegs^17^. Previous studies suggested that the elongated rete pegs in gingival overgrowth may result from increased epithelial plasticity, which leads to a phenotypic transition known as epithelial to mesenchymal transition (EMT)^18–21^. EMT is a unique process where epithelial cells undergo morphological changes that transform them from an epithelial cobblestone to a more elongated mesenchymal-like phenotype, leading to increased motility and invasion. EMT is characterized by a gradual loss of cell junction-related proteins such as E-cadherin, α E-catenin and gain of expression of mesenchymal markers such as vimentin^22,23^. In addition to its protease inhibitory function, SPOCK1 promotes tumor invasion and metastasis by inducing EMT in several cancer types, include esophageal squamous cell carcinoma^24^, lung^25^, and gastric^26^ cancers.

EMT contributes to both fibrosis and cancer progression pathologies. The initiation and progression of EMT involve distinct signaling pathways such as TGF-β1, which is a potent inducer of EMT not only through SMAD-mediated activation of EMT transcription factors^27^, but also through other signaling pathways like the PI3K/AKT pathway^28^. Indeed, SPOCK1 has been shown to induce EMT through the TGF-β1 pathway^25,29^ and was reported to exert an anti-apoptotic effect by activating the PI3K/AKT pathway^22,30–33^.

EMT involves the degradation of the basement membrane (BM) underlying epithelial cells, which leads to increased interactions between epithelial and connective tissue layers that contribute to a fibrotic pathology^19,21,34^. MMP-2 and MMP-9 are the primary MMPs responsible for BM degradation and both have reported to degrade collagen type IV which is one of the main components in the basement membrane^35,36^, and SPOCK1 has been reported to increase the expression and activity of MMP-9 in a hepatocellular carcinoma cell line^37^. Furthermore, MMP-9 was down-regulated in a *SPOCK1* knockdown, and up-regulated when *SPOCK1* was overexpressed in prostate cell lines^38^. MMP-9 plays an important role in the EMT process not only by degrading the basement membrane^39^ but also through TGF-β1 activation. TGF-β1 is secreted as an inactive multi-protein complex and MMP-9 is one of the enzymes that activates latent TGF-β1^40,41^. Some studies suggested a possible association between DIGO and EMT where decreased expression of epithelial markers such as E-cadherin and elevated expression of mesenchymal marker such as fibroblast specific protein-1 have been detected in PHE, CsA and NFD-induced gingival overgrowth tissues, and they concluded that these drugs might induce EMT possibly through TGF-β1 pathway^18,19,42,43^. However, the precise triggering molecules have not been elucidated yet. In addition, the lack of a transgenic *in vivo* model for gingival overgrowth was a limitation in those studies. Therefore, we aimed to investigate the potential triggering factors of EMT and identify the molecular mechanism for DIGO by using a transgenic mouse gingival overgrowth model.

Due to SPOCK1’s protease inhibitory function as well as its involvement in several EMT-related pathways such as AKT, TGF-β1, and MMP-9 induction, we hypothesized that SPOCK1 is a novel molecule that triggers the EMT process through its interaction with TGF-β1 and MMP-9. Here, we demonstrate for the first time that the up-regulation of SPOCK1 is associated with an EMT phenotype that accompanies gingival overgrowth in patient samples, as well as transgenic mouse model, and *in vitro* studies. Our findings suggest a novel mechanism triggered by SPOCK1 interactions with TGF-β1 and MMP-9 that induces several signaling pathways in drug-induced gingival overgrowth. This novel mechanism may assist in improving our understanding of the EMT process in non-cancerous tissues as well as the potential risk of using NFD in oral cancer patients.

## Results

### SPOCK1, TGF-β1, and MMP-9 expression levels in calcium channel blocker-induced gingival overgrowth tissues

**Figure 1:**
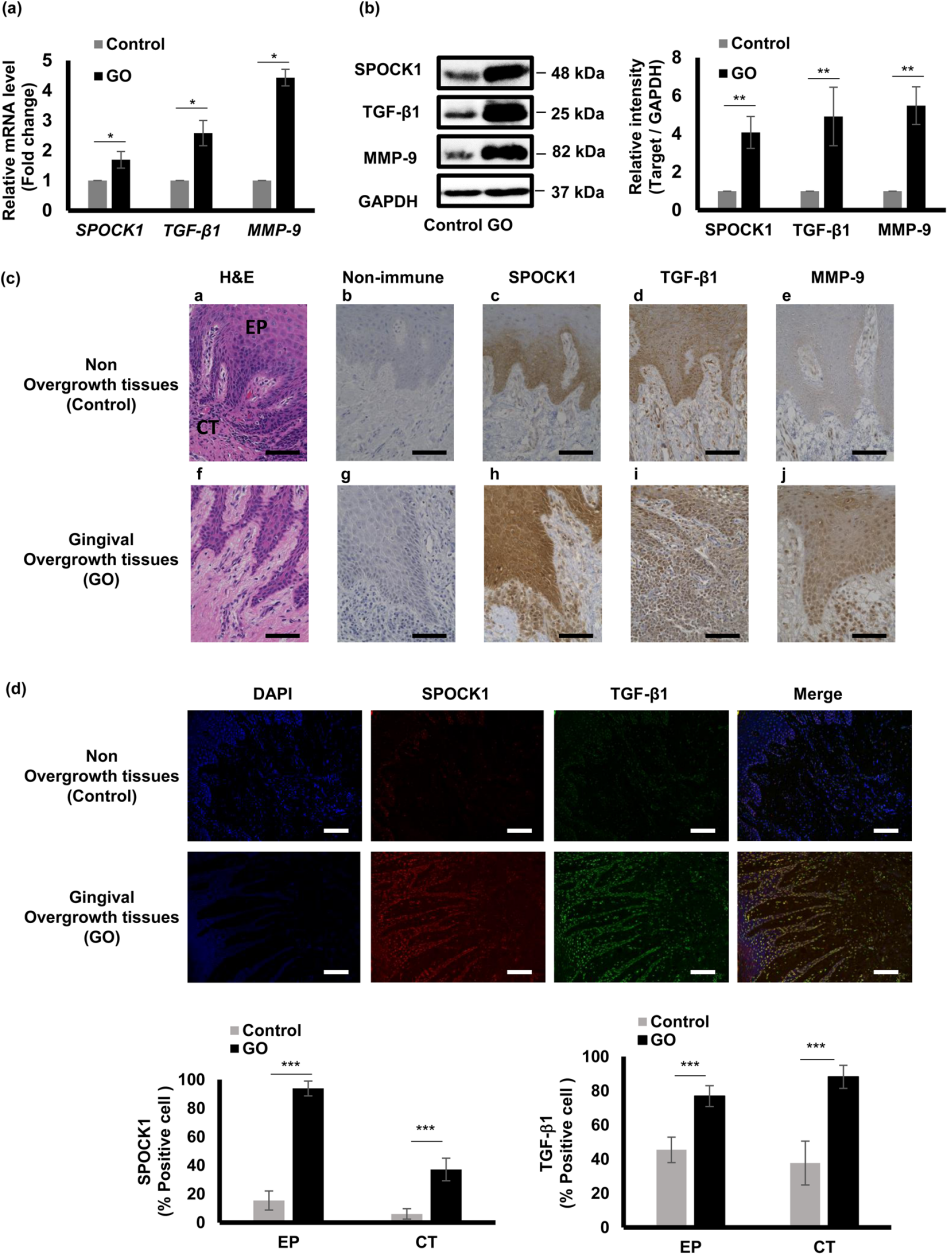
SPOCK1, TGF-β1, and MMP-9 expression levels in calcium channel blocker-induced gingival overgrowth tissues. **(a)** Relative gene expression levels of *SPOCK1*, *TGF-β1*, and *MMP-9* in gingival overgrowth (GO) and non-overgrowth (control) samples. Total RNA was extracted from homogenized gingival tissues for RT-PCR and *GAPDH* expression was used as an internal control. (**b)** Protein expression levels of SPOCK1, TGF-β1, and MMP-9 in GO and control samples determined by western blotting. (**c)** Representative sections from epithelium (EP) and connective tissue (CT) for H&E (**a, f**) and immunohistochemical staining for non-immune (**b, g**) SPOCK1 (**c, h**), TGF-β1 (**d, i**), MMP-9 (**e, j**) in control (**a, b, c, d, e**) and overgrowth (**f, g, h, i, j**) gingival tissues at x40 magnification. Scale bar = 100 μm. (**d)** Representative IF sections of SPOCK1 and TGF-β1 in control and overgrowth tissues at x40 magnification. Scale bar = 100 μm. Data are presented as mean ± SEM; *P < 0.05, **P < 0.01, ***P < 0.001 compared with controls by analysis of variance with Mann–Whitney U test (n = 4).

EMT occurs in different physiological and pathological conditions such as wound healing, tissue regeneration, organ fibrosis, and tumor progression^44^. SPOCK1, TGF-β1, and MMP-9 are associated with the EMT process in several cancer types so, we asked if these molecules are also involved in EMT-related DIGO. To answer this question, we investigated their gene and protein expression levels and location within overgrowth and non-overgrowth gingival tissues. We found that *SPOCK1*, *TGF-*β*1*, and *MMP-9* mRNA (Fig. 1a) and their protein products (Fig. 1b) were significantly higher in gingival overgrowth (GO) samples compared to non-overgrowth controls.

Next, we assessed the localization of these three molecules within the non-overgrowth and overgrowth samples by immunohistochemistry (IHC) analysis (Fig. 1c). Representative images of hematoxylin and eosin (H&E) staining showed elongated rete pegs of the epithelium (EP) that extended into the connective tissue (CT) and accumulated ECM (Fig. 1c: a,f). Non-immune control staining showed no background (Fig. 1c: b,g). SPOCK1 was strongly localized in the EP of GO samples, especially in the basal layer but less so in the connective tissue of GO samples (Fig. 1c: c,h). The opposite was found for TGF-β1, which showed intense expression in the overgrowth CT compared to the connective tissue of non-overgrowth samples (Fig. 1c: d,i). Moreover, MMP-9 was expressed more in EP and less in CT of overgrowth samples, while its expression was barely detectable in the control samples (Fig. 1c: e,j). To further confirm the expression of SPOCK1 and TGF-β1 in both epithelium and connective tissue, we also performed immunofluorescence (IF) staining. Consistent with IHC, the results showed that SPOCK1 and TGF-β1 are expressed in both epithelium and connective tissue. However, SPOCK1 was intensively expressed in the epithelium, while TGF-β1 was highly expressed within the connective tissue of the overgrowth samples (Fig. 1d).

In calcium channel blocker-induced gingival overgrowth (CCBGO) tissues; SPOCK1, TGF-β1, and MMP-9 gene and protein expression levels were significantly higher than that in non-overgrowth tissues.

### *Spock*1 transgenic mice develop gingival overgrowth and fibrosis phenotypes

**Figure 2:**
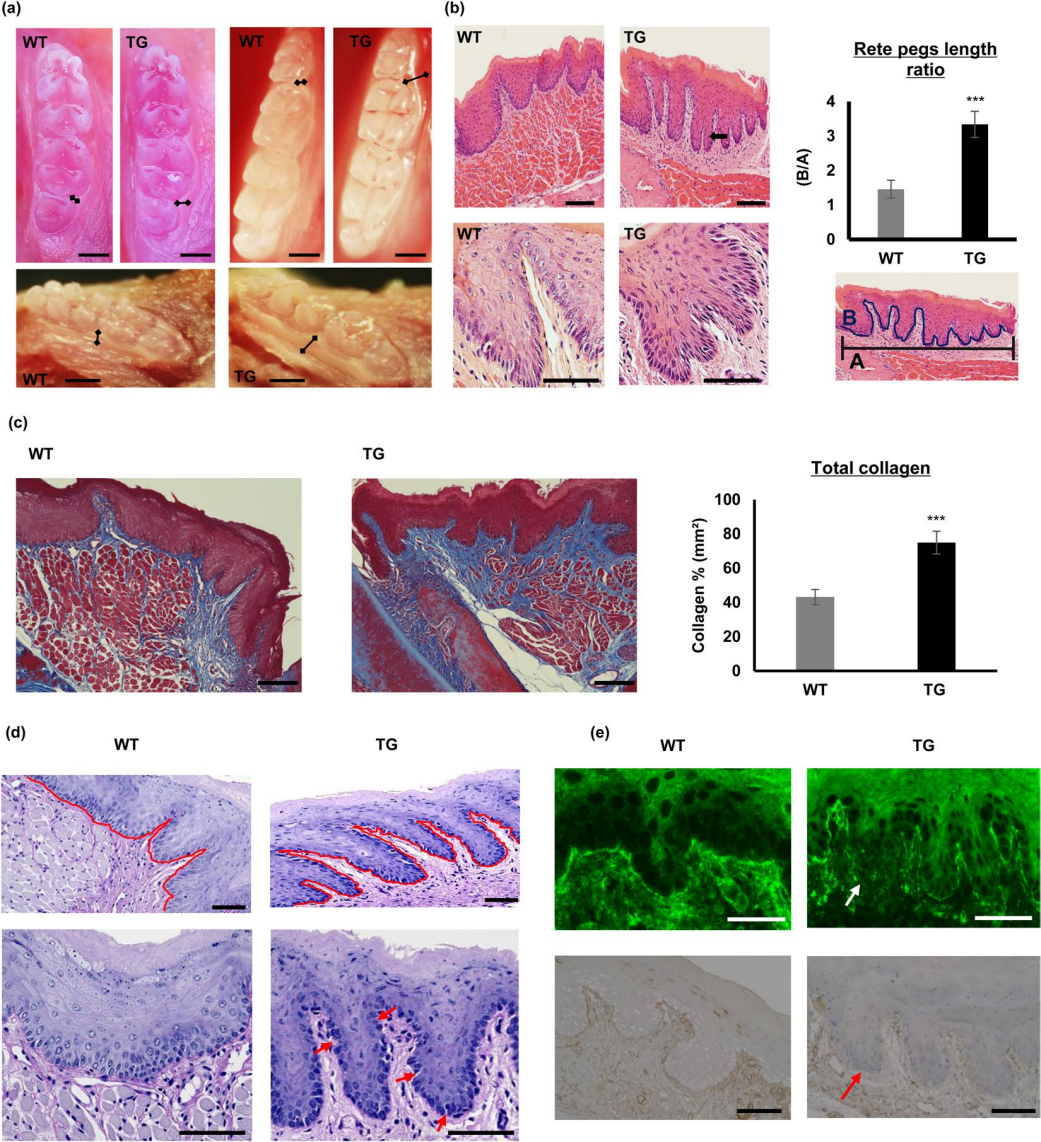
*Spock*1 TG mice developed gingival overgrowth and fibrosis phenotypes. (a) Macroscopic lingual and occlusal views of the mandibular molar region of *Spock1* TG and WT littermates. Black arrows indicate enlarged gingival tissues seen in *Spock1* TG mice at the molar region. Scale bar = 300 μm (**b)** H&E stained gingival tissues from *Spock1* TG and wild-type specimens at low magnification x20 (upper panel) that show significantly more elongated rete pegs (arrow), the total length of the basement membrane was measured (B) and normalized to a 500 μm horizontal distance measured from the cementoenamel junction (A) as shown in the illustrated image. The measurements were calculated by Image-J software, and the graph shows the ratio of the total pegs length to the horizontal length (B/A). Data are shown as means ± SEM; ***P < 0.001 compared with WT mice analyzed by Mann-Whitney U test (n = 7). Sections at higher magnification x40 (lower panel) show the difference in the appearance of the epithelial layer in TG and WT mice. (**c)** Representative images of Masson Trichrome staining of *Spock1* TG and WT mice gingiva at x20 magnification. For total collagen (blue) quantification, 12 sites with a corresponding area of 0.5 mm² for each mouse section was defined and measured by Image-J software. The graph shows the percentage of collagen staining relative to the total area. Data shown are presented as means ± SEM; ***P < 0.001 compared with WT mice analyzed by Mann-Whitney U test (n = 6), scale bar = 100 μm. (**d)** Representative images of periodic acid–Schiff (PAS) staining of *Spock1* TG and WT mice gingival BM (purple) at x40 magnification. TG mice BM exhibited irregularities (irregular red line; upper panel) compared to the more uniform BM in WT gingiva. The lower panel shows several breaks in the basement membrane in TG mice gingiva in contrast to the integral WT gingival basement membrane. Scale bar = 50 μm. **(e)** Representative images of collagen IV expression in the basement membrane shown by IHC and IF. WT gingiva shows a continuous expression of collagen IV throughout the basement membrane, while that of TG gingiva shows a weak or no expression (arrows). scale bar = 50 μm.

Based on CCBGO samples results (Fig. 1) and previous reports that SPOCK1 might induce EMT in several cancer types^24–26^, we hypothesized that SPOCK1 can induce gingival overgrowth through the EMT process. Therefore, TG mice overexpressing *Spock1* were generated (Supplementary Fig. 1) to elucidate the *in vivo* effect of *Spock1* overexpression on gingival tissue and EMT-related molecules. First, we confirmed the presence of the transgene by genotyping (genomic PCR) and the overexpression of SPOCK1 protein in gingival tissue was analyzed by western blotting (Supplementary Fig. 1). Only mice with positive SPOCK1 overexpression in the gingiva were used for subsequent experiments. Eight weeks after birth, both transgenic (TG) and wild type (WT) mice were sacrificed and mandibular dissection was performed. *Spock1* TG mice showed no obvious developmental abnormalities compared to WT mice, and the TG mice were fertile with a normal lifespan. However, upon oral cavity inspection, we detected enlarged gingival tissue in the lower molar area, especially at the buccal side in TG mice compared to the WT mice (Fig. 2a; black arrows at the lingual and occlusal views). Moreover, H&E staining indicated significantly more elongated rete pegs that extended deep into the connective tissue in TG mice gingiva, and at higher magnification we could observe dark nuclei throughout the epithelial layer in TG mice, indicating epithelial cell hyperactivity compared to the epithelial cells in WT mouse gingiva (Fig. 2b). Therefore, to check the proliferation activity in the epithelial layer, we performed IHC for the proliferation marker Ki 67, but there was no significant difference between its expression in TG and WT mice gingiva (Supplementary Fig. 1), indicating that proliferative activity was unchanged in TG mice despite the observed gingival overgrowth.

The H&E stained sections indicated that collagen fibers were densely arranged in the connective tissue of TG mice gingiva; therefore, we performed Masson Trichrome staining to evaluate total collagen accumulation within the connective tissue. The blue color after staining indicated total collagen accumulation and we found that total collagen was significantly more distributed in TG mice gingiva, especially in the epithelium-connective tissue interface, compared to WT mice gingiva (Fig. 2c).

Degradation of the basement membrane (BM) is essential for epithelial-mesenchymal transitional cells to move down into the connective tissue. To evaluate BM integrity, we performed periodic acid-Schiff (PAS) staining, which labels the BM in purple. In TG mice, gingival BM exhibited more irregularities compared to the uniform shape of the BM in WT controls (Fig. 2d; upper panel). We also observed several breaks throughout TG mice gingival sections that were sometimes surrounded by cells that were most likely transitioning cells; however, the WT gingival sections had continuous labeling throughout the tissue sections (Fig. 2d; lower panel). Since collagen IV is a main component of the BM, and MMP-9 is reported to break-down collagen IV^35,36^, we performed both IHC and IF to check collagen IV expression. The results showed a continuous expression of collagen IV in the BM of the WT mice when compared to a weak or no expression in some part of the BM in the TG mice gingiva (Fig. 2e).

Collectively, we found that *Spock1* TG mice manifested overgrowth with extended rete pegs accompanied by accumulated collagen within the connective tissue and the BM showed multiple breaks throughout the tissue sections with decreased collagen IV expression.

### SPOCK1 overexpression induces EMT-like changes at the histological and molecular levels in mice gingiva

**Figure 3:**
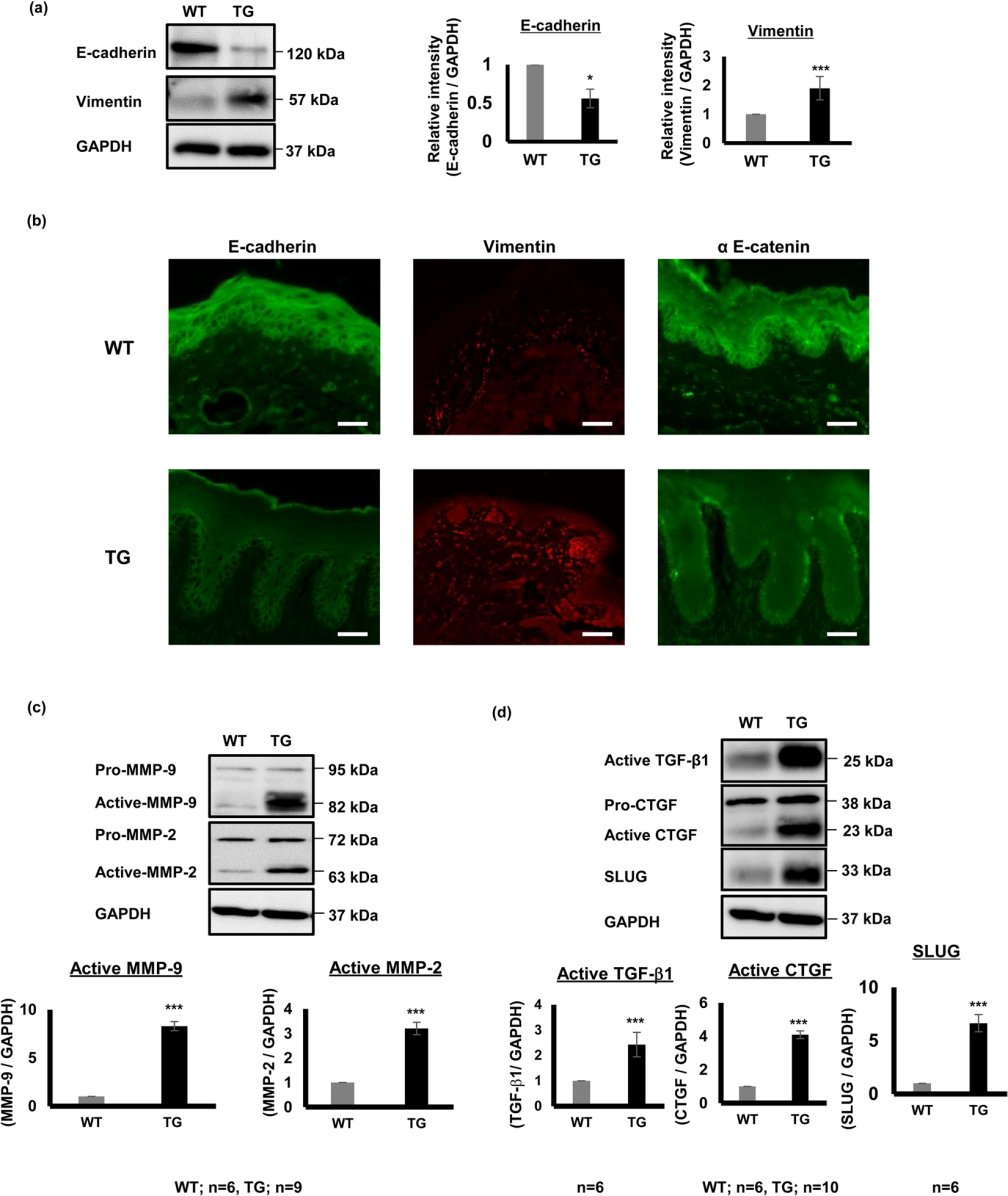
SPOCK1 overexpression induces EMT-like changes at histological and molecular levels in mice gingiva. **(a)** Relative protein expression of E-cadherin and vimentin was assessed by western blotting in TG and WT mice gingival tissues with GAPDH as an internal control. Data shown are presented as means ± SEM; *P < 0.05 and ***P < 0.001 compared with WT controls analyzed by the Mann-Whitney U test, (n = 5 per group). (**b)** IF staining of E-cadherin, vimentin and α E-catenin of TG and WT specimens at x40 magnification. The cells in the epithelium express less E-cadherin and α E-catenin, while cells within the connective and the basal layer of the epithelium expressed more vimentin in TG mouse gingiva compared to WT gingiva. scale bar = 100 μm. (**c)** Relative protein expression levels of MMP-9 and MMP-2 were measured by western blotting in TG and WT mouse gingiva, with GAPDH used as an internal control. Data shown are presented as means ± SEM; ***P < 0.001 compared with WT controls analyzed by the Mann-Whitney U test, (WT n = 6, TG n = 9). (**d)** Relative protein expression of TGF-β1, CTGF, and SLUG measured by western blotting in TG and WT mouse gingiva. GAPDH was used as internal control. Data shown are presented as means ± SEM; ***P < 0.001 compared with WT controls analyzed by the Mann-Whitney U test.

We next investigated the protein expression of molecules related to the EMT process such as E-cadherin, α E-catenin and vimentin in mice gingiva. Using western blotting, we evaluated the epithelial marker E-cadherin because its down-regulation is a hallmark of EMT, as well as the mesenchymal marker vimentin. As expected, E-cadherin expression level was significantly down-regulated and vimentin expression was significantly up-regulated in TG mice gingival tissues compared to WT mouse gingiva (Fig. 3a). In IF sections, E-cadherin and α E-catenin were mainly localized in the epithelium of WT mice gingiva, while their expression levels were reduced in TG mice gingiva. Additionally, cells within the connective tissue and some cells in the basal layer of the epithelium expressed more vimentin in TG mice gingiva when compared with that in the WT controls (Fig. 3b).

Because we detected breaks in the basement membrane by PAS staining and lower expression of collagen IV in TG mice gingiva (Fig. 2d, e) we investigated the expression of both MMP-9 and MMP-2, which are the main enzymes responsible for BM degradation including collagen IV. Both active MMP-9 and MMP-2 expression levels were up-regulated in TG mice gingival tissue compared to WT, but there was no significant difference in the pro-form of these two enzymes (Fig. 3c). We next investigated the other EMT-related molecules, TGF-β1, CTGF, and SLUG.

TGF-β1 is a potent inducer of EMT and its downstream molecule CTGF is an inducer of extracellular matrix production. The active forms of both molecules were significantly up-regulated in TG mice gingiva compared to WT controls. Finally, we examined the expression of SLUG, a transcription factor responsible for E-cadherin suppression, and found it was up-regulated in TG mice gingival tissues compared with the WT controls (Fig. 3d).

### The effect of NFD on SPOCK1, TGF-β1, and MMP-9 expression levels in gingival keratinocytes and fibroblasts

**Figure 4:**
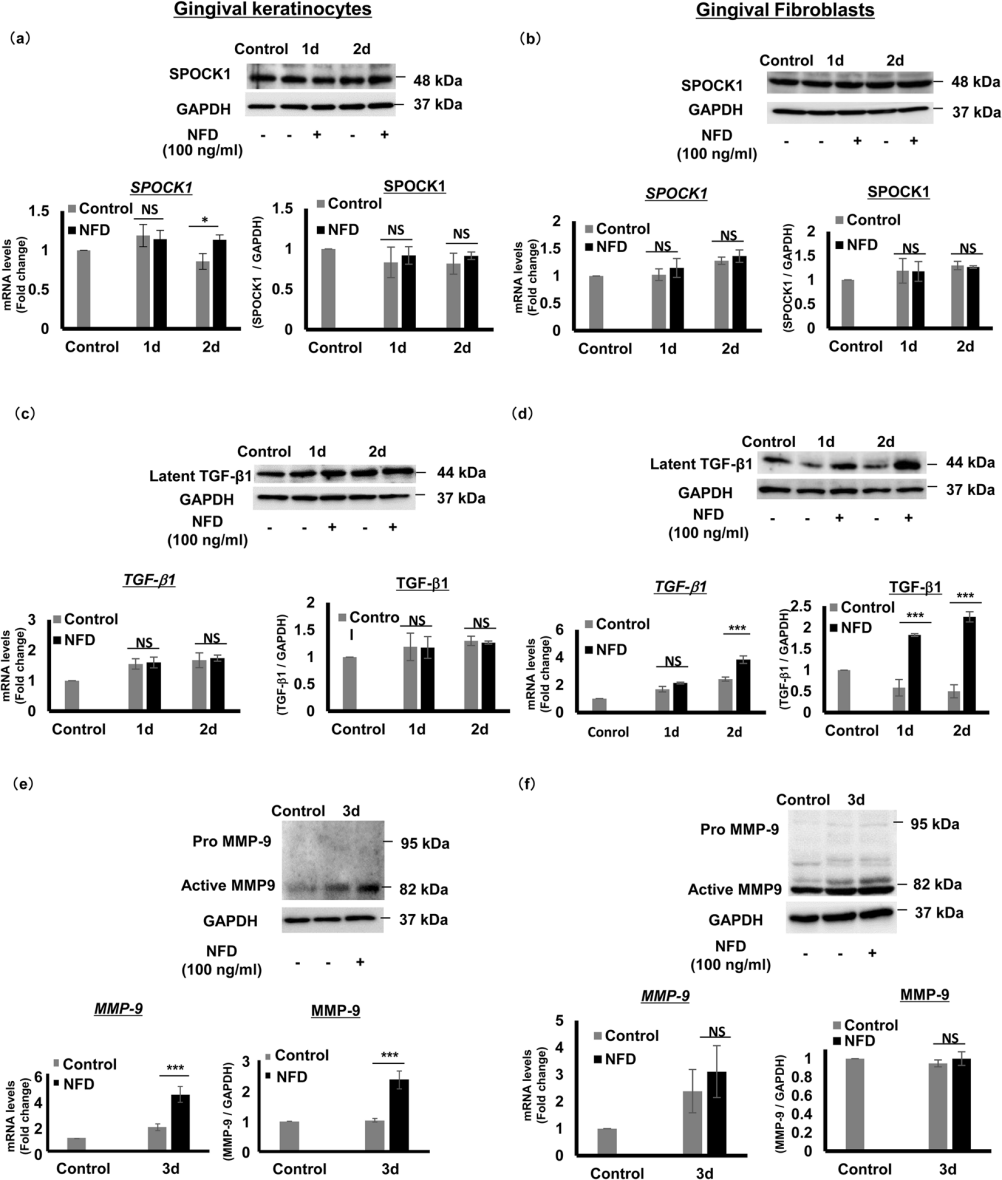
The effect of NFD on SPOCK1, TGF-β1, and MMP-9 expression levels in gingival keratinocytes and fibroblasts. Gene and protein expression levels of SPOCK1, TGF-β1, and MMP-9 in MOE1a (**a,c,e**), and primary human gingival fibroblasts (HGF) (**b,d,f**) by RT-PCR and western blotting. Relative SPOCK1 mRNA and protein levels in (**a**) MOE1a cells stimulated with 100 ng/mL NFD for 1 and 2 days or (**b**) in primary HGF stimulated with 100 ng/mL NFD for 1 and 2 days. Relative latent TGF-β1 mRNA and protein expression levels in (**c**) MOE1a cells stimulated with 100 ng/mL NFD for 1 and 2 days; and (**d**) primary HGF cells stimulated with 100 ng/mL NFD for 1 and 2 days. Relative MMP-9 mRNA and protein expression levels in (**e**) MOE1a cells stimulated with 100 ng/mL NFD for 3 days; and (**f**) primary HGF stimulated with 100 ng/mL NFD for 3 days. Immunoblot data are representative of three independent experiments with similar results. Gene expression analyses were performed at least three times with triplicate samples. Data are presented as mean ± SEM; NS; not significant, *P < 0.05, ***P < 0.001 compared with unstimulated controls by analysis of variance with Tukey’s test (n = 3).

In patient samples, we detected the up-regulation of SPOCK1, TGF-β1, and MMP-9 in either epithelium or connective tissue or both; therefore, we asked if NFD would have a similar effect on these molecules *in vitro*. Both a mouth-ordinary-epithelium (MOE1a) human gingival keratinocyte cell line and primary human gingival fibroblasts (HGF) were stimulated with NFD for 1 and 2 days, but no significant difference in SPOCK1 mRNA and protein expression levels were observed in either MOE1a (Fig. 4a) or primary HGF (Fig. 4b). However, although there was no significant difference in latent TGF-β1 mRNA and protein expression levels in the MOE1a cell line (Fig. 4c), in primary HGF, the mRNA levels were significantly up-regulated on the second day of NFD stimulation and the protein level was up-regulated on both days of stimulation compared to the unstimulated controls (Fig. 4d). Unlike TGF-β1, the mRNA and protein expression levels of active MMP-9 were significantly up-regulated 3 days after stimulation in MOE1a cells (Fig. 4e), while there was no significant difference in active MMP-9 mRNA and protein expression levels in primary HGF cells compared to unstimulated controls (Fig. 4f). MMP-9 is known to degrade the BM, and also activate TGF-β1 along with MMP-2, MMP-13, and MMP-14, all of which were unaffected by NFD treatment at the mRNA expression level (Supplementary Fig. 2). Although NFD did not appear to affect SPOCK1 gene and protein expression levels in both gingival keratinocytes and fibroblasts, it did cause the up-regulation of latent TGF-β1 in gingival fibroblasts and active MMP-9 in gingival keratinocytes.

### The effects of CsA and PHE on TGF-β1 and SPOCK1 in primary human fibroblasts

**Figure 5:**
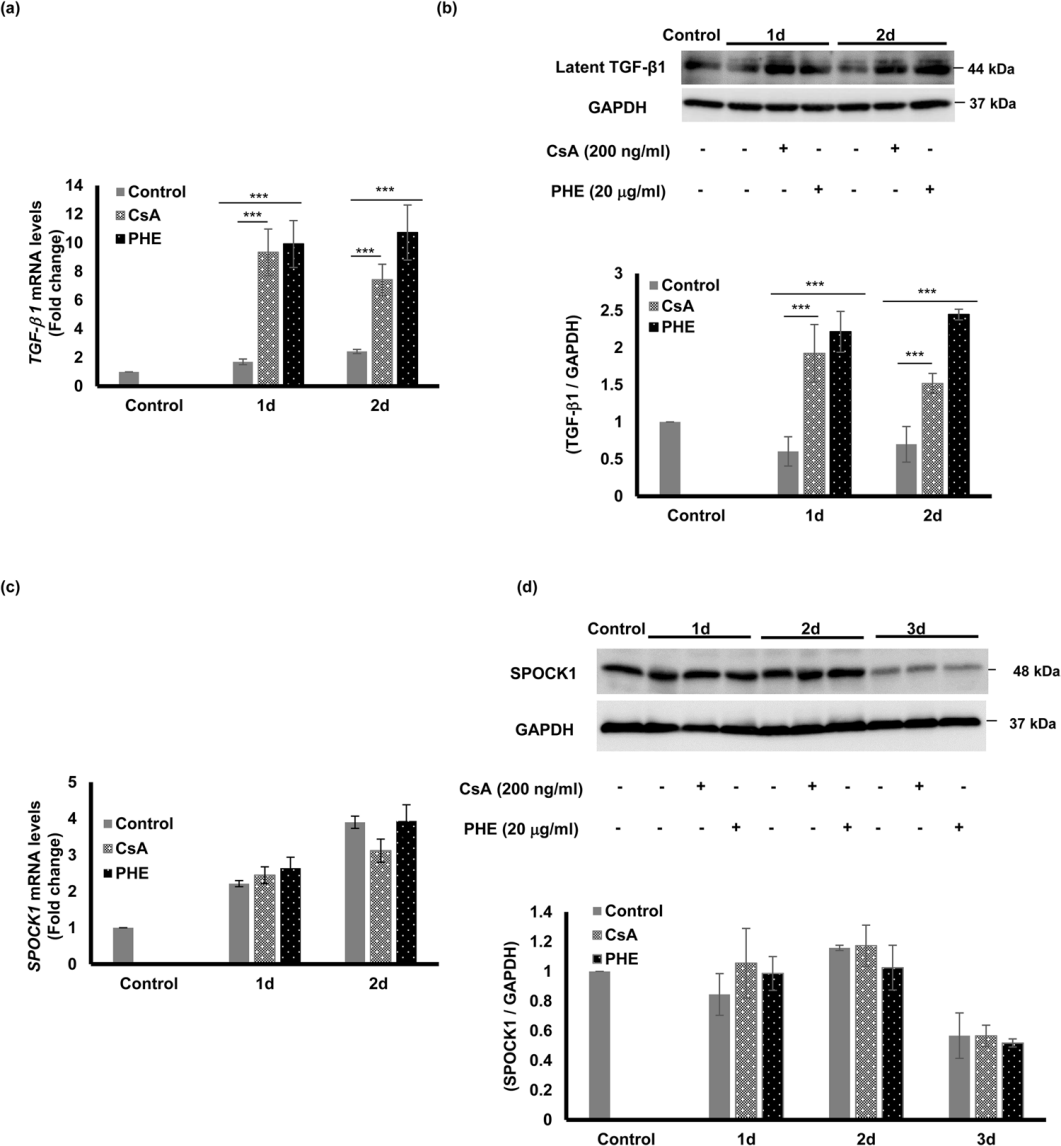
The effects of CsA and PHE on TGF-β1 and SPOCK1 expression in primary human gingival fibroblasts. **(a)** Relative latent *TGF-β1* mRNA expression level in primary human gingival fibroblasts (HGF) stimulated with 200 ng/mL CsA and 20 μg/mL PHE for 1 and 2 days. Total RNA was extracted and real time RT-PCR was conducted using *GAPDH* as an internal control. (**b)** Relative latent TGF-β1 protein expression level in HGF stimulated with 200 ng/mL CsA and 20 μg/mL PHE for 1 and 2 days. Total protein was extracted and analyzed by western blotting using GAPDH protein expression as an internal control. Immunoblot data are representative of three independent experiments with similar results. **(c)** Relative *SPOCK1* mRNA expression level in primary human gingival fibroblasts (HGF) stimulated with 200 ng/mL CsA and 20 μg/mL PHE for 1 and 2 days. Total RNA was extracted and real time RT-PCR was conducted using *GAPDH* as an internal control. (**d)** Relative SPOCK1 protein expression level in HGF stimulated with 200 ng/mL CsA and 20 μg/mL PHE for 1, 2 and 3 days. Total protein was extracted and analyzed by western blotting using GAPDH as an internal control. Gene expression analyses were performed at least three times with triplicate samples. Data are presented as mean ± SEM; ***P < 0.001 compared with unstimulated controls by analysis of variance with Tukey’s test (n = 3).

TGF-β1 is a potent inducer of EMT in epithelial cells^25,29^. Upon TGF-β1 stimulation in MOE1a cells, *E-cadherin* mRNA expression levels were significantly down-regulated, while *vimentin*, *MMP-2*, *MMP-9*, and *SLUG* were significantly up-regulated compared with unstimulated controls (Supplementary Fig. 3). NFD induced the up-regulation of latent TGF-β1 in HGF; therefore, we asked if CsA and PHE, which are well-known drugs that induce gingival overgrowth, would have a similar effect on TGF-β1. We stimulated HGF with 200 ng/mL CsA and 20 μg/mL PHE for 1 and 2 days and found that both drug treatments significantly up-regulated latent TGF-β1 mRNA (Fig. 5a) and protein levels (Fig. 5b) compared to the control conditions. Since there observed no direct effect on SPOCK1 expression when HGF was stimulated with NFD (Fig. 4b), we wondered if similar results would be obtained when HGF was stimulated with CsA and PHE. Thus, we checked SPOCK1 gene and protein expression and confirmed that SPOCK1 levels were unchanged by these causative drugs (Fig. 5c,d).

### The effect of TGF-β1 on SPOCK1 in gingival keratinocytes and fibroblasts

**Figure 6:**
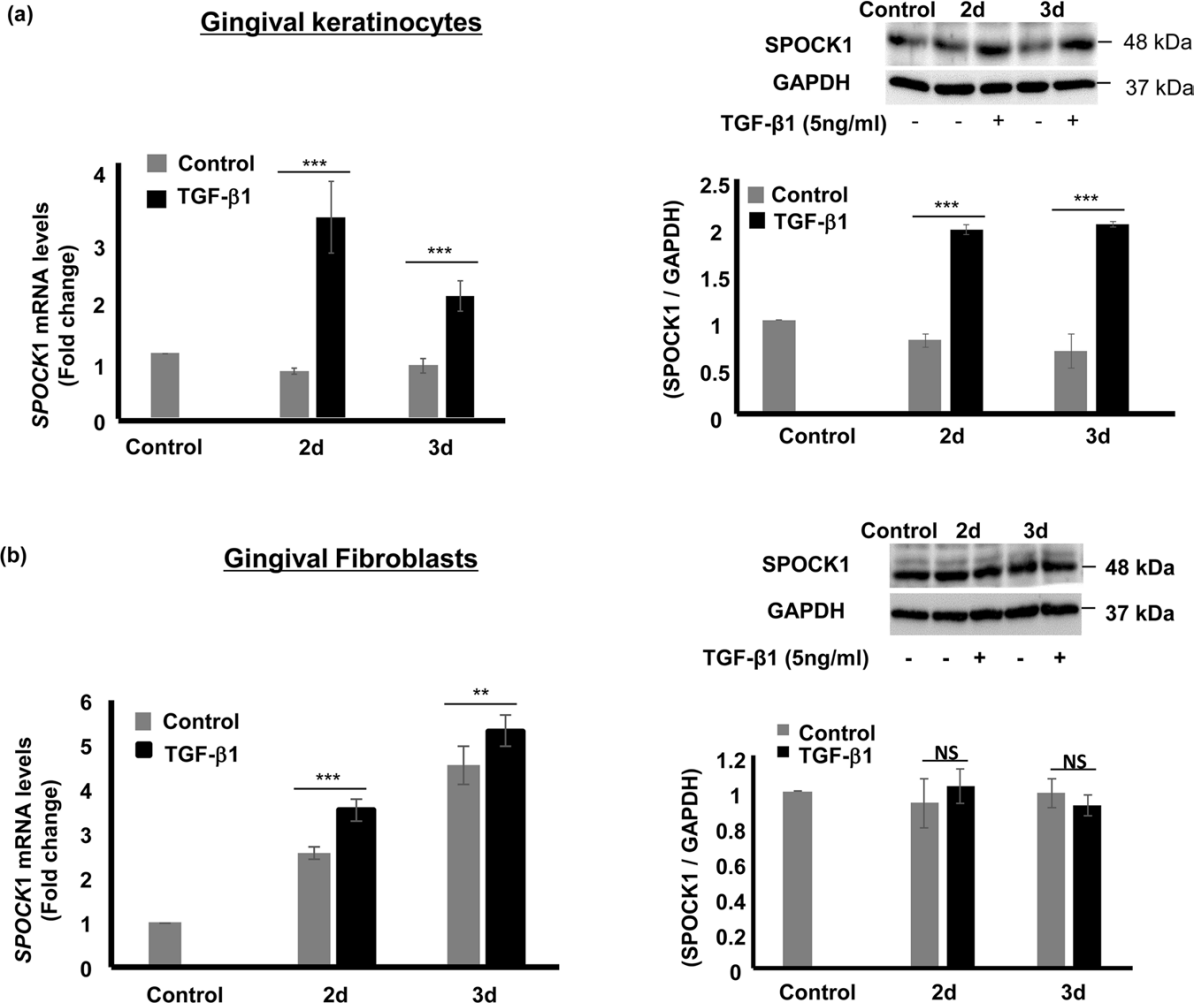
The effect of TGF-β1 on SPOCK1 expression in gingival keratinocytes and fibroblasts. Gene and protein expression levels of SPOCK1 in MOE1a and primary human gingival fibroblasts were measured by RT-PCR and western blotting, respectively. (**a)** Relative SPOCK1 mRNA and protein expression levels in MOE1a cells stimulated with 5 ng/mL TGF-β1 for 2 and 3 days. (**b)** Relative SPOCK1 mRNA and protein expression levels in primary HGF stimulated with 5 ng/mL TGF-β1 for 2 and 3 days. Immunoblot data are representative of three independent experiments with similar results. Gene expression analyses were performed at least three times with triplicate samples. Data are presented as mean ± SEM; NS; not significant, **P < 0.01, ***P < 0.001 compared with unstimulated controls by analysis of variance with Tukey’s test (n = 3).

Although SPOCK1 was up-regulated in CCBGO samples; however, *in vitro* NFD, CsA and PHE did not have a direct effect on SPOCK1 expression. Therefore, we hypothesized that there was an indirect effect of these drugs on SPOCK1 expression during gingival overgrowth. Because SPOCK1 is a downstream target of TGF-β1, we investigated the effect of TGF-β1 stimulation in MOE1a and primary HGF. We found that SPOCK1 mRNA and protein levels were significantly up-regulated in MOE1a (Fig. 6a), but that TGF-β1 stimulation only slightly up-regulated *SPOCK1* mRNA expression and had no effect on its protein level in HGF (Fig. 6b). Although it is well known that TGF-β1 induces SPOCK1 gene and protein expression levels, this effect seems limited to keratinocytes only.

## Discussion

Drug-induced gingival overgrowth (DIGO) is tissue-specific and occurs in approximately half of the people treated with the anti-seizure medication phenytoin, anti-hypertensive drug nifedipine, and immune suppressor cyclosporin A, which are widely and commonly prescribed^45^. There are similar histopathological characteristics of all forms of DIGO, which led to the assumption that these drugs, despite their differences, act through a common mechanism^3^. Although DIGO has been investigated extensively, its exact mechanism is not fully understood. Many reports focused on DIGO in either epithelium or connective tissue; however, in the current study we propose an interactive model between epithelial and connective tissues that contribute to DIGO development through the EMT process. Although EMT is implicated in DIGO, the main inducer of EMT remains unknown. SPOCK1 is a secreted proteoglycan reported to induce EMT in several cancer cell lines and up-regulate the expression and activity of MMP-9 ^24–26,37,38^. In addition, TGF-β1 is a strong inducer of EMT and fibrosis, and MMP-9 is reported to activate TGF-β1^40,41^ as well as degrade BM, which is necessary for the migration of epithelial cells to underlying connective tissue. Therefore, we investigated both the functional relationships between SPOCK1, TGF-β1, and MMP-9 and their role in inducing EMT in gingival overgrowth using *in vitro* and *in vivo* models.

In CCBGO samples, we detected the up-regulation of both gene and protein levels of SPOCK1, TGF-β1, and MMP-9 (Fig. 1). In the current study, SPOCK1 was strongly expressed within the epithelium and less so in the underlying stromal layer, which was consistent with the SPOCK1 expression pattern observed in cancerous lesions in head and neck squamous cell carcinoma^46^. Along with the up-regulation of SPOCK1, MMP-9, and TGF-β1 were also up-regulated in overgrowth samples. Based on this observation, we hypothesized that SPOCK1 induces the EMT process by interacting with TGF-β1 and MMP-9. To further investigate our hypothesis, we generated a TG mouse overexpressing *Spock1* model to study the functional relationships between these molecules and other EMT markers. Although *Spock1* knockout mice showed no development or gross phenotype^47^, *Spock1* overexpression TG mice did develop an overgrowth and fibrosis in their gingival tissue (Fig. 2). First, compared to control WT mice, we detected different degrees of overgrowth in the gingival tissues of the lower molar area in TG mice more prominently in the buccal side. We also detected more elongated rete pegs and increased collagen accumulation similar to that observed in human gingival overgrowth. Moreover, in this transgenic model, clear irregularities at the epithelium and connective tissue interface and multiple breaks in the basement membrane accompanied by decreased collagen IV expression were observed. These observations may indicate degradation of the BM and acquired epithelial plasticity that led to the extension of the epithelial pegs into the connective tissue. These results are consistent with results from drug-induced gingival overgrowth tissues that exhibit discontinuities in basement membranes accompanied by a decrease in both collagen type IV and laminin 5 expression in the BM^21^.

We further investigated EMT-related molecules in TG mice gingiva. We initially confirmed increased epithelial plasticity due to E-cadherin and α E-catenin down-regulation and up-regulation of vimentin in connective tissue, which could explain the elongated rete pegs. The active forms of MMP-2 and MMP-9 “the main enzymes in BM degradation”^48^ were up-regulated in *Spock1* TG mice gingiva. However, there was no change in their pro-forms, indicating that SPOCK1 overexpression only affected the activity of MMP-9 and MMP-2. In a previous study, MMP-9 was up-regulated by SPOCK1 in a hepatocellular carcinoma cell line and treatment with an MMP-9 inhibitor significantly inhibited the invasion ability of SPOCK1^37^, suggesting SPOCK1 may induce ECM remodeling through MMPs.

TGF-β1 plays an important role in fibrosis by stimulating synthesis of collagen in the lamina propria. Connective tissue growth factor (CTGF) is a downstream target of TGF-β1 signaling^19^. A previous study showed that levels of CTGF are highest in phenytoin, intermediate in nifedipine, and very low in cyclosporin A-induced gingival overgrowth lesions^20^. CTGF sustains fibrosis initiated by TGF-β1^49^ and thus, neither of the two factors alone are able to initiate fibrotic processes^50^. Because of the high level of active CTGF and TGF-β1 in *Spock1* TG mice gingiva, we detected more ECM accumulation, especially collagen in the connective tissue (Fig. 2c). At the transcription level, a genetic switch is needed to facilitate the loss of epithelial markers and the gain of mesenchymal markers. SLUG is a transcription repressor of E-cadherin whose expression was found to be significantly high in TG mice compared to WT mice (Fig. 3d). In line with our findings, a previous study showed that SPOCK1 facilitated EMT in gastric cancer through the activation of SLUG rather than SNAIL^26^. Because SPOCK1 was mainly expressed in the epithelium of TG mice gingiva, especially in the basal layer, we asked if SPOCK1 overexpression would affect the proliferation of basal gingival epithelial cells. To do this, we performed Ki 67 staining to evaluate changes in basal gingival epithelial cell proliferation in the *Spock1* TG and WT mouse gingiva. Although there was no difference in epithelial cell activity between TG and WT mice, it is possible that SPOCK1 might not affect epithelial cell proliferative activity but rather, it might diminish keratinocyte programmed death that occurs in several cancers through the activation of PI3K/AKT signaling that blocks apoptosis^30,32,37,51^.

In DIGO, some reports show that rather than increased keratinocyte proliferation, decreased apoptosis had a more prominent role in the pathogenesis of gingival overgrowth^39,52–54^. To investigate the effect of NFD *in vitro*, we used primary HGF and the non-cancerous gingival keratinocyte cell line MOE1a to ensure that the effect of NFD would reflect the real effect of the drug on native keratinocytes and fibroblasts. Unlike other keratinocytes that are immortalized by human papilloma virus, MOE1a cells maintain the characteristics of normal epithelial cells without acquiring typical features of cancer cells, including EMT phenotypes^55^. To our knowledge, the effect of NFD, CsA, and PHE on SPOCK1 has never been investigated before and in this study, although these drugs had no direct effect on SPOCK1 expression in keratinocytes or fibroblasts, we hypothesized that the up-regulation of SPOCK1 is mediated by another molecule, such as TGF-β1. Indeed, NFD, CsA, and PHE up-regulated the latent TGF-β1 expression level in fibroblasts (Figs. 4 and 5) and TGF-β1 caused the up-regulation of SPOCK1 in gingival keratinocytes (Fig. 6). In our study, we also showed that NFD increased latent TGF-β1 in fibroblasts and increased active MMP-9 in the keratinocytes, which could activate TGF-β1 to induce SPOCK1 expression in keratinocytes in a paracrine manner.

Taken together, we propose an interactive model to explain the pathogenesis of DIGO in which SPOCK1, TGF-β1, and MMP-9 interact to form a tri-directional regulatory loop that induces EMT. In our model, the interaction between EP and CT is an essential element for inducing gingival overgrowth. NFD has a dual role in stimulating both fibroblasts to produce latent TGF-β1 and keratinocytes to produce active MMP-9. TGF-β1 is proteolytically activated by MMP-9 to exert its biological functions, which include a paracrine effect that stimulates SPOCK1 expression in keratinocytes. Moreover, SPOCK1 increases MMP-9 activity that degrades the BM and activates latent TGF-β1. As a result of BM degradation and the gradual acquirement of the mesenchymal phenotype, the epithelial “transition cells” start migrating deep into the connective tissue where they contribute to the production of ECM within the stroma. This model is consistent with the observed elongated rete pegs and the accumulated ECM, which are the two primary features of DIGO.

**Figure 7:**
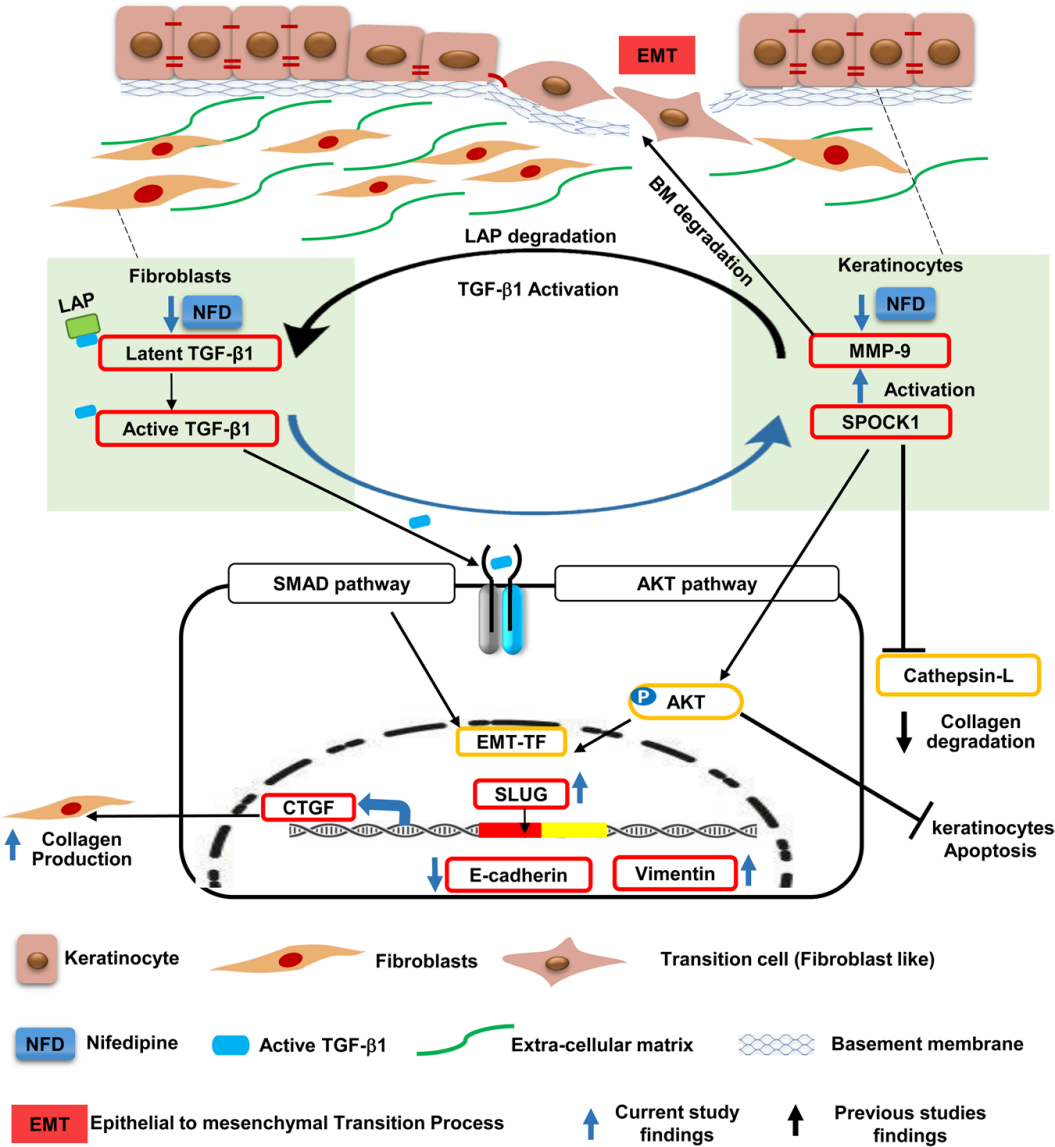
Proposed novel model of the pathogenesis of drug-induced gingival overgrowth. Schematic illustration of the interaction between SPOCK1, TGF-β1, and MMP-9 in epithelial and connective tissues that induces gingival overgrowth. This novel model explains the elongated rete pegs and the accumulated extracellular matrix, all of which are main characteristic features of drug induced gingival overgrowth.

Our *in vitro* and *in vivo* data strongly suggest that SPOCK1 plays a key role in inducing DIGO through cooperation and crosstalk between translation regulation and signaling pathways. First, SLUG induction by SPOCK1 could be the main switch that causes cells to acquire a migratory phenotype by inhibiting E-cadherin expression. Second, PI3K/AKT pathway induction not only has anti-apoptotic effects^30,32,37,51^in epithelial cells but also induces the EMT process through up-regulation of transcription factors such as SLUG^56,57^. Third, TGF-β1 pathway induction and subsequent CTGF production leads to an increase in ECM production within connective tissue. Fourth, SPOCK1 in the connective tissue may exert a protease inhibitory effect on proteases such as Cathepsin L^15,16^ that leads to the inhibition of ECM degradation within connective tissue (Fig. 7).

Collectively, our studies indicate SPOCK1 could be a main trigger of gingival overgrowth and potential therapeutic target for DIGO treatment. Indeed, some recent reports showed that several miRNAs interact with the *SPOCK1* gene to suppress its expression^46,58^ and therefore, utilization of these miRNAs could be a therapeutic strategy for DIGO treatment through *SPOCK1* suppression. Another very important finding from our study is that NFD might induce EMT through TGF-β1-mediated SPOCK1 up-regulation, which could drive hypertensive patients with oral cancer toward a higher risk of metastasis, as EMT is one of the main mechanisms of cancer metastasis with poor prognosis^59^. Therefore, caution should be taken when treating hypertension with NFD in subjects with oral cancer.

The reported tri-directional interaction between SPOCK1, TGF-β1, and MMP-9 during DIGO induction could be further investigated by 3D culture system to see the actual interaction of keratinocytes, fibroblasts and extra-cellular matrix components. For future studies, it would be interesting to see the effect of drug administration on the transgenic mice gingiva, and another uncovered aspect in our study is the possible association between SPOCK1 overexpression and inflammation. Previous studies indicated CsA or PHE are enough to induce the overgrowth and inflammation exacerbated the degree of gingival overgrowth^60,61^. Therefore, it would be worth investigating the combined effects of SPOCK1 overexpression and inflammation induced by ligature-model or so on.

In conclusion, our study showed for the first time that SPOCK1 up-regulation induces EMT in a pathological condition other than cancer through several possible pathways and points to SPOCK1 as a novel therapeutic target for DIGO.

## Experimental Procedures

All methods described below were carried out in accordance with relevant guidelines and regulations of the Journal.

### Patient samples

Calcium channel blocker-induced gingival overgrowth and non-overgrowth gingival tissue samples were obtained from patients attending Kyushu University Hospital (Supplementary Table 1). Following tissue excision, tissues were either fixed in 4% paraformaldehyde solution (Nacalai Tesque, Kyoto, Japan) and embedded in paraffin or homogenized in TRIzol (Thermo Fisher Scientific, MA, USA) or CytoBuster Protein Extraction Reagent (Millipore, Billerica, MA, USA) for RT-PCR or western blotting, respectively. Immunohistochemical staining (IHC) was performed using primary antibodies against SPOCK1 primary antibody (1:100; #MAB2327 R&D SYSTEMS, Minnesota, USA), TGF-β1 (1:100; #ab92486 Abcam, Cambridge, UK), and MMP-9 (1:100; #sc-21733 Santa Cruz Biotechnology, Dallas, USA). Serial sections were stained with non-immune IgG isotype as negative controls to ensure no background staining. Heat-induced epitope retrieval was performed in a citrate buffer (pH 9.0 or pH 6.0) according to the manufacture instructions. The sections were incubated with primary antibodies overnight at 4 °C and treated with a biotin-free horseradish peroxidase enzyme-labeled polymer (EnVision, + /HRP; Dako Cytomation, Gostrup, Denmark) for 30 min at room temperature. The sections were then treated with a 3,3′-diaminobenzidine tetrachloride substrate solution and counterstained with hematoxylin. The samples were visualized by (BZ-9000, Keyence BZ-X Analyzer, Osaka, Japan).

### Mice

*Spock1* overexpression transgenic (TG) mice were generated by Trans Genic Inc (Kobe, JAPAN). The structure of the pCAGGS-Spock1-Myc expression vector is shown in Supplementary Fig. 1. The pUC57-Spock1-myc construct was synthesized with the Kozak and Myc-tag coding sequences. Th*e Spock1* cDNA sequence was inserted between Nhe I and Xho I sites in the pCAGGS vector. The transgene was cut from the vector with Asc I, Hind III and ApaL I restriction enzymes and microinjected into 200 fertilized C57BL/6 N mouse eggs to generate founder mice (F0). The DNA was extracted from founder mice tail samples with KAPA Express Extract (Kapa Biosystems, MA, USA) for genotyping using the primer pairs:

pCAGGS-F1: 5′-GTCGACATTGATTATTGACTAGTTATTAAT-3′

pCAGGS-R2275: 5′-GTCGAGGGATCTCCATAAGAGAAGAGGGACA-3′

TG founder mice were crossed with WT C57BL/6 N mice to generate F1 TG mice. Gingival tissues were analyzed by western blotting to confirm SPOCK1 protein over-expression. The F2 TG offspring mice were generated by crossing F1 TG mice with WT (C57BL/6 N) mice. Their descendants were used in the experiments, followed by confirmation of the presence of *Spock1* transgene by genotyping and SPOCK1 protein overexpression in the gingival tissues by western blotting (Supplementary Fig. 1b,c). Eight weeks after birth, male *Spock1* (TG) mice and male wild type (WT) littermates were sacrificed and mandibles were dissected. First, the mandibular gingival tissues were observed under a microscope (BX 50; Olympus, Tokyo, Japan), then the right mandibles were fixed in 4% paraformaldehyde solution (Nacalai Tesque), decalcified in 20% formic acid solution at room temperature overnight, and embedded in paraffin. The gingival tissues from left mandibles were used for western blotting.

### Immunofluorescence staining and analysis

IF staining was performed as previously described^24^. Briefly, paraffin embedded tissues of both patient and mice samples were cut into 3 μm sections and they were dewaxed in Xylene and rehydrated in graded ethanol solutions. Antigen retrieval was performed with citrate buffer (6 pH) for 10 minutes at 95 °C. Sections rinsed in PBS for 5 min. Non-specific labeling was blocked by incubation with 1% bovine serum albumin at room temperature for 30 min. Sections were then incubated with SPOCK1 mouse anti-human (1:200; R&D SYSTEMS), TGF-β1 rabbit anti-human (1:200; Abcam) Collagen IV rabbit anti-mouse (2:200; Abcam, #ab6568), E-cadherin rabbit anti-mouse (1:200; Cell Signaling Technology, Beverly, MA, #24E10), vimentin mouse anti-mouse (4:200; Santa Cruz), andαE-catenin rabbit anti-mouse (6:200; Proteintech, #12831-1-AP) primary antibodies at 4 °C overnight, the sections washed and incubated with Alexa 488-conjugated goat anti-rabbit IgG (1:200; Abcam, #ab150077) or Alexa 647-conjugated goat anti-mouse IgG (1:200; Abcam, #ab150115). The nucleus stained with DAPI (Invitrogen, Thermo Fisher Scientific, #s36968). The samples were visualized by Keyence BZ-9000. All patient samples images were analyzed by Keyence BZ-X Analyzer software, where the numbers of the labelled cells were counted in two randomly selected areas in each sample (n = 4) and normalized to the total number of the cells presented in each sections in both EP and CT tissue.

### Histological analysis

Sections 3-μm thick were cut from paraffin-embedded mouse gingival tissues. Sections were stained with hematoxylin and eosin (H&E), periodic acid-Schiff (PAS), and Masson Trichrome dye for histological, basement membrane, and collagen accumulation analysis, respectively. Immunohistochemistry (IHC) was performed using Collagen IV rabbit anti-mouse (1:50; Abcam, #ab6568) and Ki 67 (1:400, #122025, Cell Signaling) primary antibodies. Stained images were visualized using (BZ-9000, Keyence BZ-X Analyzer).

To evaluate the length of rete pegs in both WT and TG gingiva, the H&E stained sections were visualized at x20 and images were analyzed by image J software (National Institute of Health, Bethesda, MA). Two sections were evaluated per sample, the total length of the basement membrane was measured and normalized to a fixed 500 μm horizontal distance measured from the cementoenamel junction.

Total collagen was evaluated in the samples with Masson Trichrome dye at 12 sites with a corresponding area of 0.5 mm² that were defined and used for quantitative analysis with Image J software (National Institute of Health). All paraffin embedding and staining procedures were performed at Pathophysiological and Experimental Pathology, Department of Pathology, Graduate School of Medical Sciences, Kyushu University.

### Cell lines and cell culture

MOE1a (mouth-ordinary-epithelium) is a non-cancerous human gingival epithelial cell line generated at the Department of Biochemistry and Genetics, Kagoshima University Graduate School of Dental and Medical Sciences [51]. The MOE1a cells were maintained in 1X defined keratinocyte-SFM medium (Gibco, Thermo Fisher Scientific, Waltham, MA, USA) at 37 °C and 5% CO_2_. The cells were sub-cultured as previously described [51] and stimulated with 100 ng/mL nifedipine (#141–05783, Wako, Odawara, Japan) and 5 ng/mL recombinant human TGF-β1 (#AF-100, Pepro Tech. New Jersey, USA) when the cells reached 80% confluence. Gingival tissues were collected from patients attending the Periodontology Department at Kyushu University Hospital. The tissue samples were collected in 5 mL decontamination solution (500 mL PBS with 10 mL penicillin-streptomycin mixed solution (#26253–84, Nacalai Tesque) and 10 mL penicillin-streptomycin-amphotericin B suspension (#161-23181, Wako) the samples were then trimmed into 5 ×5 mm pieces and placed into a 35-mm dish (Corning Laboratory Science Company, New York, USA). The trimmed tissues were kept upside down in the dishes in an incubator for 10 min without medium to allow complete adhesion to the bottom of the dish. The small explants were maintained in a primary culture medium of Dulbecco’s modified Eagles medium (DMEM; Nacalai Tesque) with 15% fetal bovine serum (FBS; Biowest, FL), 2.5 g/500 mL HEPES (#7365-45-9, Dojindo Laboratories) 2% non-essential amino acid solution (Gibco, Thermo Fisher Scientific), 5 mL/500 mL penicillin-streptomycin mixed solution (#26253-84, Nacalai Tesque), and 5 mL/500 mL penicillin-streptomycin-amphotericin B suspension (#161-23181, Wako). The explants were incubated at 37 °C in 5% CO_2_ and the medium was changed every 2 days. After 2 weeks the cells started to grow out of the explants and were transferred and expanded after trypsinization. The medium was then changed to DMEM (Nacalai Tesque) containing 10% FBS (Biowest, FL). The cells were used between the 3rd and 4th passages and stimulated with 100 ng/mL nifedipine (#141- 05783, Wako), 200 ng/mL cyclosporin A (#031-24931, Wako), 20 μg/mL phenytoin (#166-12082, Wako) and 5 ng/mL recombinant Human TGF-β1 (#AF-100, Pepro Tech., New Jersey, USA).

### Real-time polymerase chain reaction (PCR)

Real-time PCR was performed as described previously^62^. Briefly, total RNAs were isolated using TRIzol reagent (Invitrogen), followed by complementary deoxyribonucleic acid (cDNA) synthesis using Prime Script RT Master Mix (Takara Bio, Shiga, Japan) according to the manufacturer’s instructions. Real-time PCR was performed using KAPA SYBR (Kapa Biosystems, Roche Sequencing, Pleasanton, CA) according to the manufacturer’s instructions. The reactions and subsequent analysis were performed using Step One Plus (Life Technologies, Thermo Fisher Scientific). Relative mRNA levels were normalized to *GAPDH* mRNA levels. The relative expression levels were determined by the comparative Ct method. Primer sequences are shown in (Supplementary Table 2).

### Western blot analysis

Either cell or tissue lysates were used for western immunoblot analysis. The samples were solubilized with CytoBuster™ Protein Extraction Reagent (Millipore, Billerica, MA, USA). Equal amounts of protein from lysates were resolved by SDS-PAGE. The proteins were then transferred to polyvinylidene difluoride membranes (Millipore) using the semi-dry system (Trans-Blot SD Semi-Dry Transfer Cell (Bio-Rad Laboratories, Hercules, CA, USA)). Primary antibodies used were as follows: anti-mouse against human SPOCK1 (R&D SYSTEMS 1:1000), anti-mouse against mouse SPOCK1 (Santa Cruz, 2:1000), anti-rabbit against human TGF-β1 (Abcam, 1:1000), anti-mouse TGF-β1 (Santa Cruz, 1:1000), anti-mouse against human MMP-9 (Santa Cruz, 2:1000), anti-mouse MMP-9 (Santa Cruz, 2:1000), anti-mouse E-cadherin (Santa Cruz, 2:1000), anti-mouse vimentin (Santa Cruz, 2:1000), anti-mouse CTGF (Santa Cruz, 2:1000), anti-mouse MMP-2 (Santa Cruz, 2:1000), anti-mouse SLUG (Santa Cruz, 2:1000), and anti-mouse GAPDH (Cell Signaling Technology, MA, USA). Samples were then incubated with secondary HRP-conjugated anti-rabbit, and anti-mouse antibodies (Cell Signaling Technology, Inc.) and visualized using enhanced chemiluminescence (Chemi-Lumi One Super, Nacalai Tesque). Signals were analyzed using Image Quant LAS4000 (GE Healthcare, Chalfont, UK). Reaction intensity was calculated using Image J software (National Institute of Health)

### Statistical analysis

All experiments were performed in triplicate. Data are expressed as the mean ± standard deviation of three independent experiments. Statistical comparisons from two groups were performed using the Mann–Whitney U test, and for multiple comparisons an analysis of variance (ANOVA) with Tukey’s HSD test was performed. All analyses were conducted in PASW SPSS Statistics 18 (Chicago, USA). We considered P values of ≤0.05 statistically significant.

### Study approval

Collection of patient samples and their use in this study were approved by Kyushu University Institutional Review Board for Clinical Research (No. 30-372). Written informed consent was obtained from all patients prior to all procedures. All animal experiments were performed in accordance with Guides for the Care and Use of Laboratory Animals and with the approval of the Kyushu University Animal Experiment Committee (No. A19-145-1).

## Supplementary information

Supplementary information

## Data Availability

The datasets generated and/or analyzed during the current study are available from the corresponding author on reasonable request.
