# Supplementary material for: SPOCK1 is a novel inducer of epithelial to mesenchymal transition in drug-induced gingival overgrowth
Source: Sci Rep. 2020 Jun 17;10:9785. doi: 10.1038/s41598-020-66660-z (PMC7300011; doi:10.1038/s41598-020-66660-z)

Supplementary Information

SPOCK1 is a novel inducer of epithelial to mesenchymal transition in drug-induced gingival overgrowth

Rehab Alshargabi[1], Tomomi Sano[1], Akiko Yamashita[1], Aiko Takano[1], Taiki Sanada[1], Misaki Iwashita[1], Takanori Shinjo[1], Takao Fukuda[1], Terukazu Sanui[1], Shosei Kishida[2], Fusanori Nishimura[1]

Affiliations of the authors

1 - Section of Periodontology, Division of Oral Rehabilitation, Kyushu University Faculty of Dental Science, Fukuoka, Japan.

3-1-1 Maidashi, Higashi-ku, Fukuoka, 812-8582, Japan.

2 - Department of Biochemistry and Genetics, Kagoshima University Graduate school of Dental and Medical Sciences, Kagoshima, Japan.

8-35-1 Sakuragaoka, Kagoshima, 890-8544, Japan.

Supplementary information:

Supplementary Fig. 1 *Spock1* transgenic mouse generation

Supplementary Fig. 2 The effect of NFD on TGF-β1 activating molecules in gingival keratinocytes

Supplementary Fig. 3 The effect of TGF-β1 on EMT markers in gingival keratinocytes

Supplementary Table 1 Calcium channel blocker-induced gingival overgrowth (GO) and non-overgrowth (Control) patient profiles

Supplementary Table 2 Primer sequences used for real-time PCR

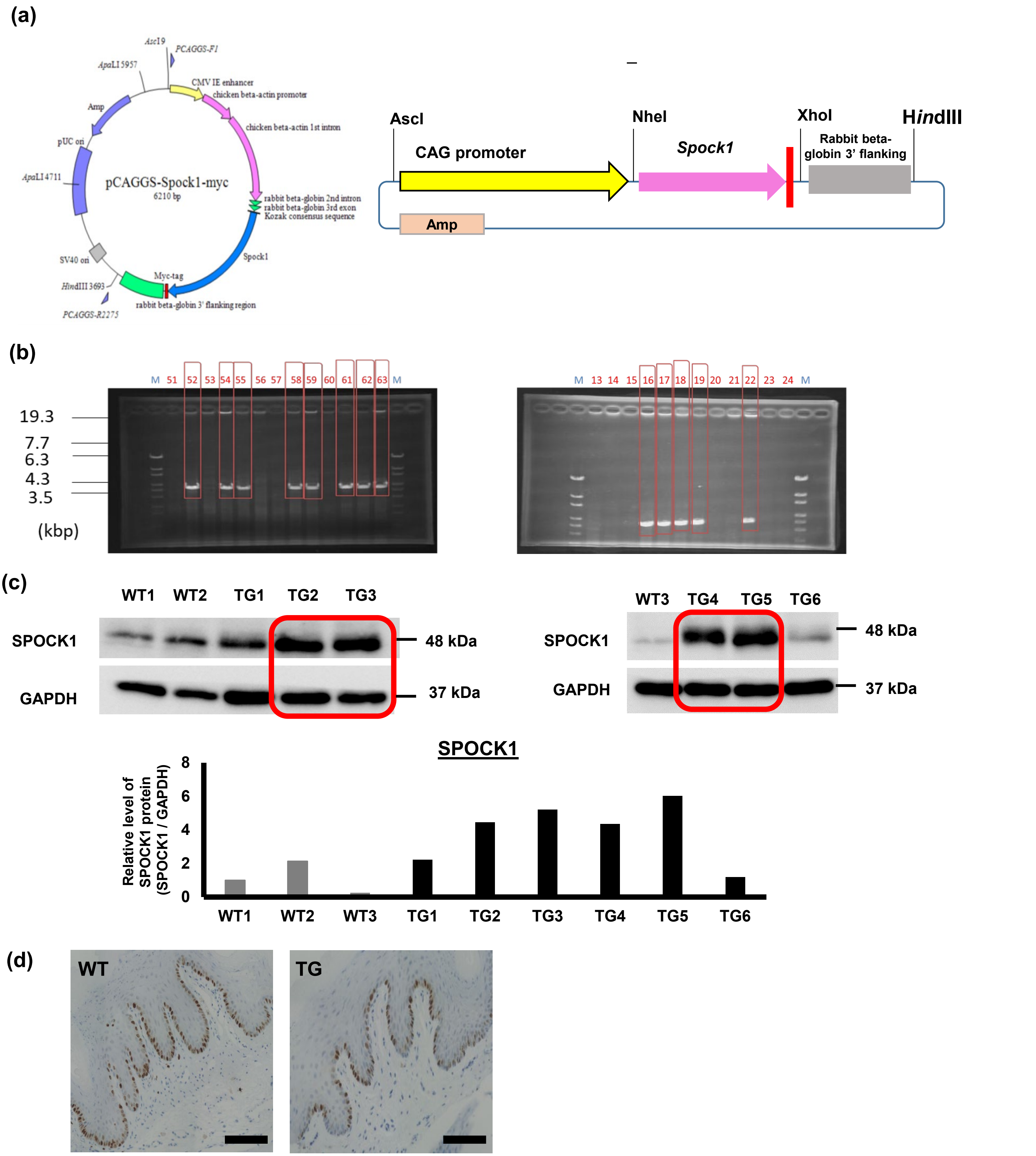

Supplementary Fig. 1 *Spock1* overexpression transgenic mouse generation

(**a**) The structure of the pCAGGS-Spock1-Myc expression vector generated Trans Genic Inc (Fukuoka, JAPAN).

(**b**) DNA was extracted from founder mice tail samples to confirm the presence of the transgene by genotyping.

(**c**) TG mice and wild type mouse gingival tissues were analyzed by western blotting to confirm SPOCK1 protein over-expression.

(**d**) Representative sections from epithelium and connective tissue used for immunohistochemical staining of Ki 67. Scale bar = 100 μm at x10 magnification.

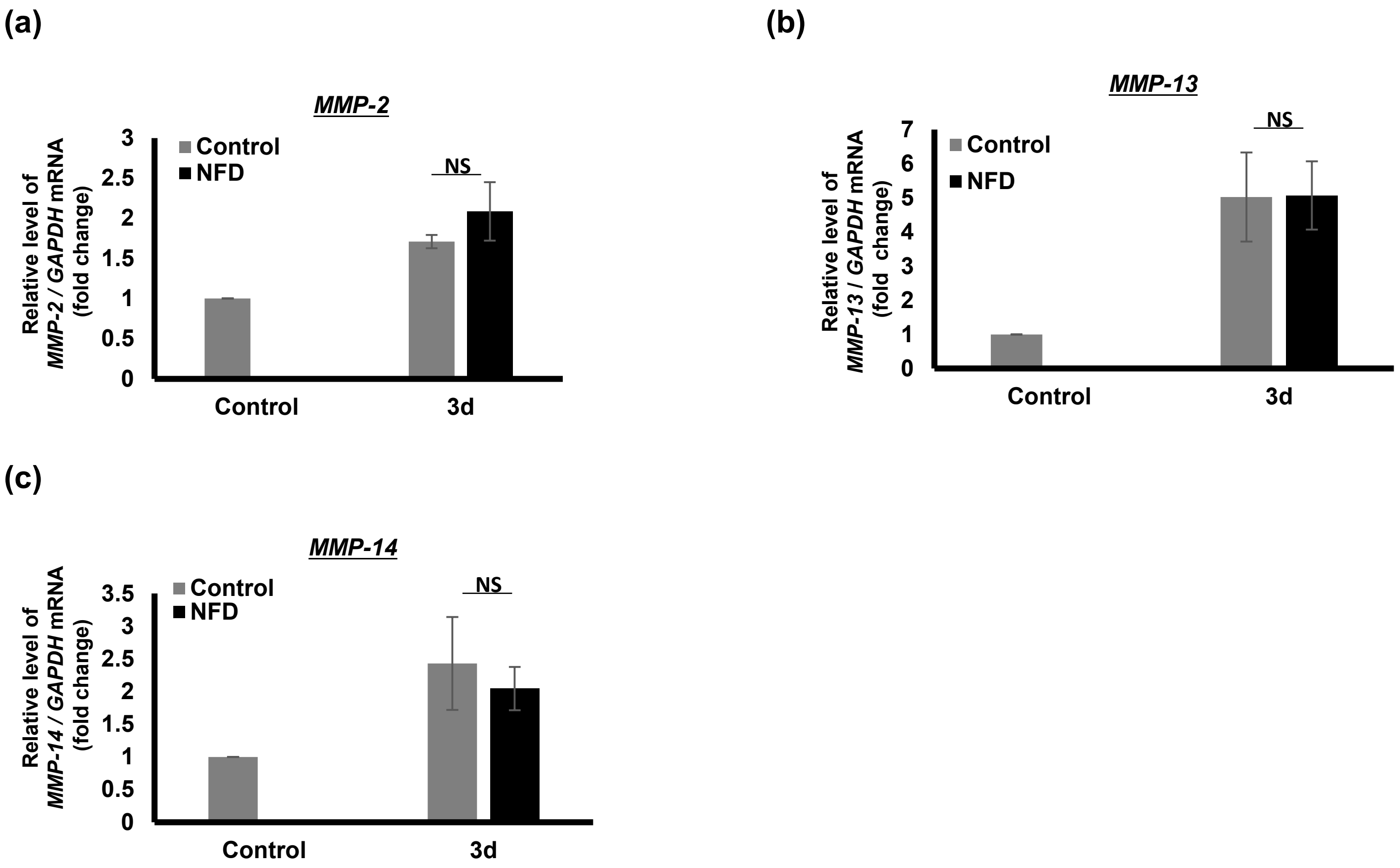

Supplementary Fig. 2 The effect of NFD on TGF-β1 activating molecules in gingival keratinocytes

Relative mRNA expression levels of (**a**) *MMP-2*; (**b**) *MMP-13*; and (**c**) *MMP-14* in MOE1a cells stimulated with NFD for three days. *GAPDH* expression was used as an internal control for real-time RT-PCR analysis. Gene expression analyses were performed at least three times with triplicate samples. Data are mean ± SEM; (NS: not significant) compared with unstimulated controls by analysis of variance with Tukey's test (n=3).

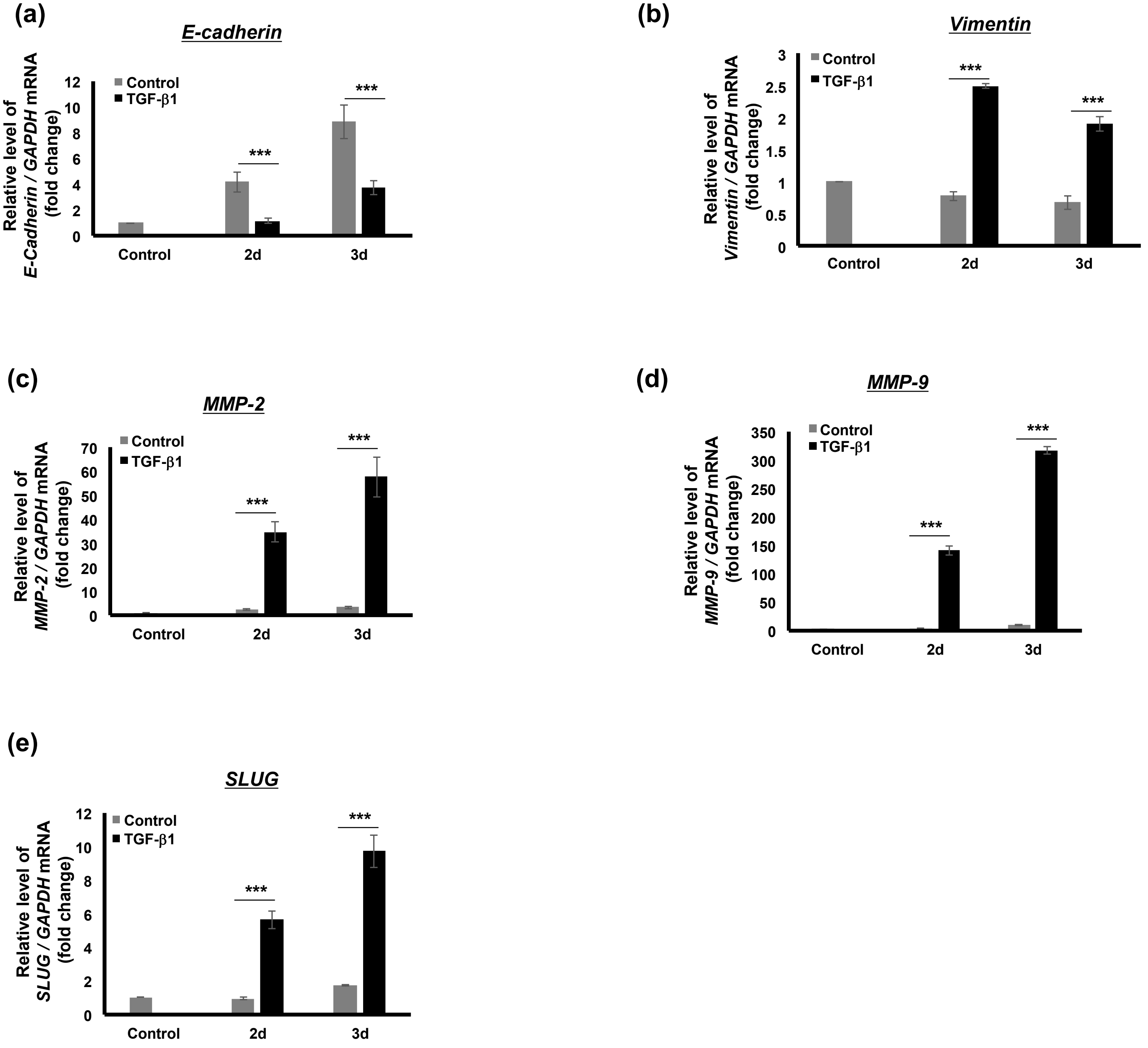

Supplementary Fig. 3 The effect of TGF-β1 on EMT markers in gingival keratinocytes

Gingival keratinocytes (MOE1a) were stimulated with 5 ng/ml TGF-β1 for 2 and 3 d before total RNA was extracted and real-time PCR was conducted using GAPDH expression as an internal control to evaluate changes in EMT-related molecules. Relative mRNA expression levels of (**a**) *E-cadherin*; (**b**) *vimentin*; (**c**) *MMP-2*; (**d**) *MMP-9*; and (**e**) *SLUG.* Gene expression analyses were performed at least three times with triplicate samples. Data are mean ± SEM; ***P< 0.001 compared with unstimulated controls by analysis of variance with Tukey's test (n=3).

Supplementary Table 1:
Calcium channel blocker-induced gingival overgrowth (GO), and non-overgrowth (Control) patient profiles:

Gingival overgrowth (GO) patients	Gender	Age	Drug consumption period
**GO1**	Male	45	Nifedipine+Amelodipine 5 yrs
**GO2**	Male	77	Amelodipine 15~20 yrs
**GO3**	Female	74	Nifedipine 30 yrs
**GO4**	Male	68	Amelodipine 5~6 yrs

Non Gingival overgrowth (control) patients	Gender	Age	Diagnosis
**Control 1**	Male	44	Chronic periodontitis
**Control 2**	Female	54	Chronic periodontitis
**Control 3**	Female	35	Chronic periodontitis
**Control 4**	Male	56	Chronic periodontitis

Supplementary Table 2

Primer sequences used for real-time PCR

Gene	Forward	Reverse
SPOCK1	GGACCCATCCAAGGACCC	GGCTTGCACTTGACCAAATTC
TGF-β1	AGCGACTCGCAAGCGTGGTTA	GCAGTGTGTTATCCCTGCTGTCA
MMP-9	ACCTCGAACTTTGACAGCGACA	GATGCCATTCACGTCGTCCTTA
MMP-2	GATAACCTGGATGCCGTCGTG	CAGCCTAGCCAGTCGGATTTG
E-cadherin	GCCGAGAGCTACACGTTCAC	ACTTTGAATCGGGTGTCGAG
Vimentin	TGCAGGAGGAGATGCTTCAG	ATTCCACTTTGCGTTCAAGG
MMP-13	TCCCAGGAATTGGTGATAAAGTAGA	CTGGCATGACGCGAACAATA
MMP-14	GAGCTCAGGGCAGTGGATAG	GGTAGCCCGGTTCTACCTTC
SLUG	CATGCCTGTCATACCACAAC	GGTGTCAGATGGAGGAGGG
GAPDH	ATCAAGAAGGTGGTGAAGCAGG	GTCATACCAGGAAATGAGC

The original images for western blot and the target panels were marked in red boxes.

Figure 1

(b)

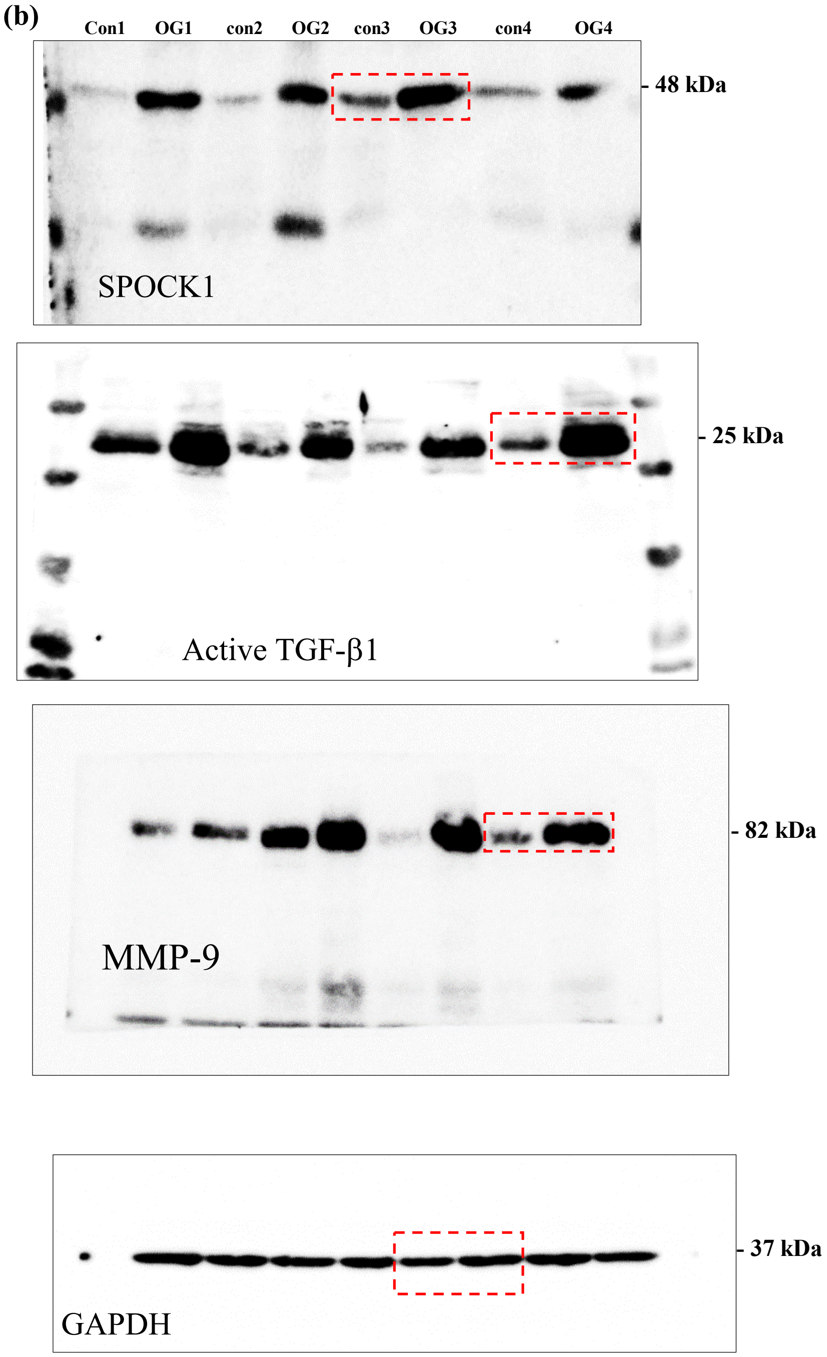

Figure 3

(a)

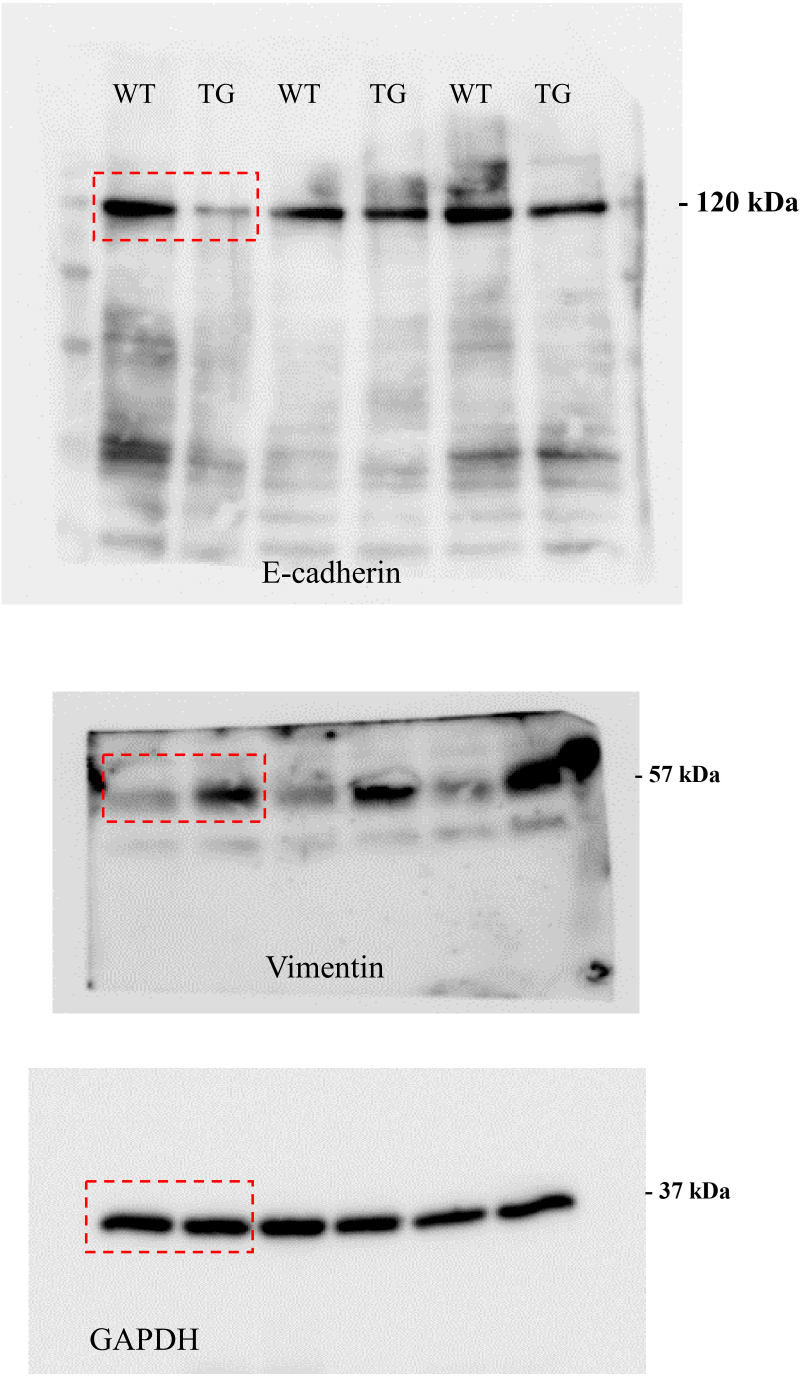
WT
TG
WT
TG
WT
TG
- 120 kDa
E-cadherin
- 57 kDa
Vimentin
- 37 kDa
GAPDH

Figure 3

(c)

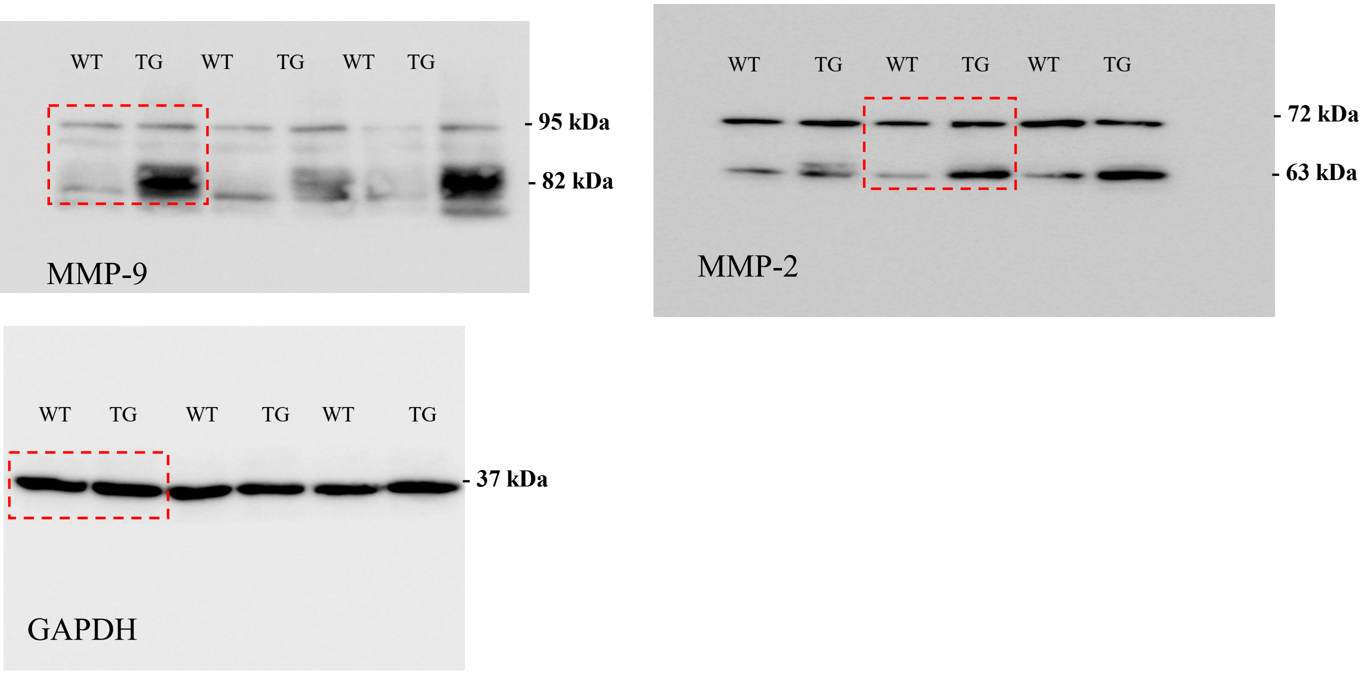
WT TG WT TG WT TG
- 95 kDa
- 82 kDa
MMP-9
WT TG WT TG WT TG
- 72 kDa
- 63 kDa
MMP-2
WT TG WT TG WT TG
- 37 kDa
GAPDH

(d)

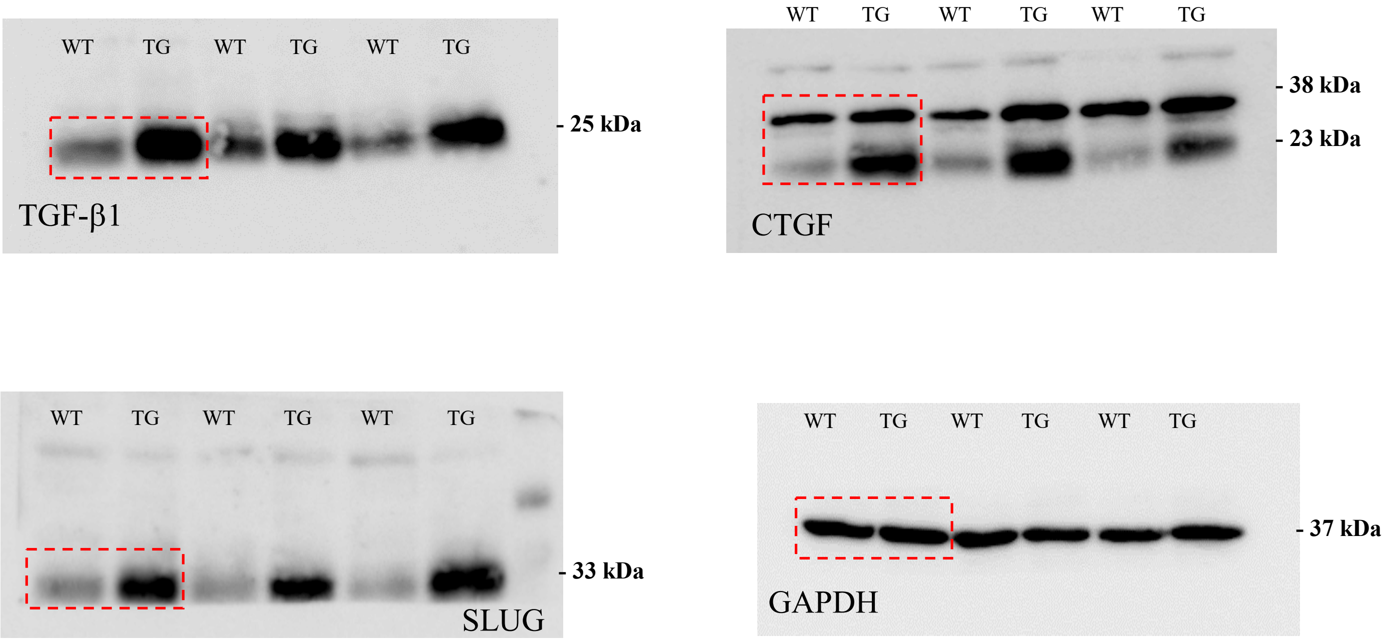
WT TG WT TG WT TG
- 25 kDa
TGF-β1
WT TG WT TG WT TG
- 38 kDa
- 23 kDa
CTGF
WT TG WT TG WT TG
- 33 kDa
SLUG
WT TG WT TG WT TG
- 37 kDa
GAPDH

Figure 4

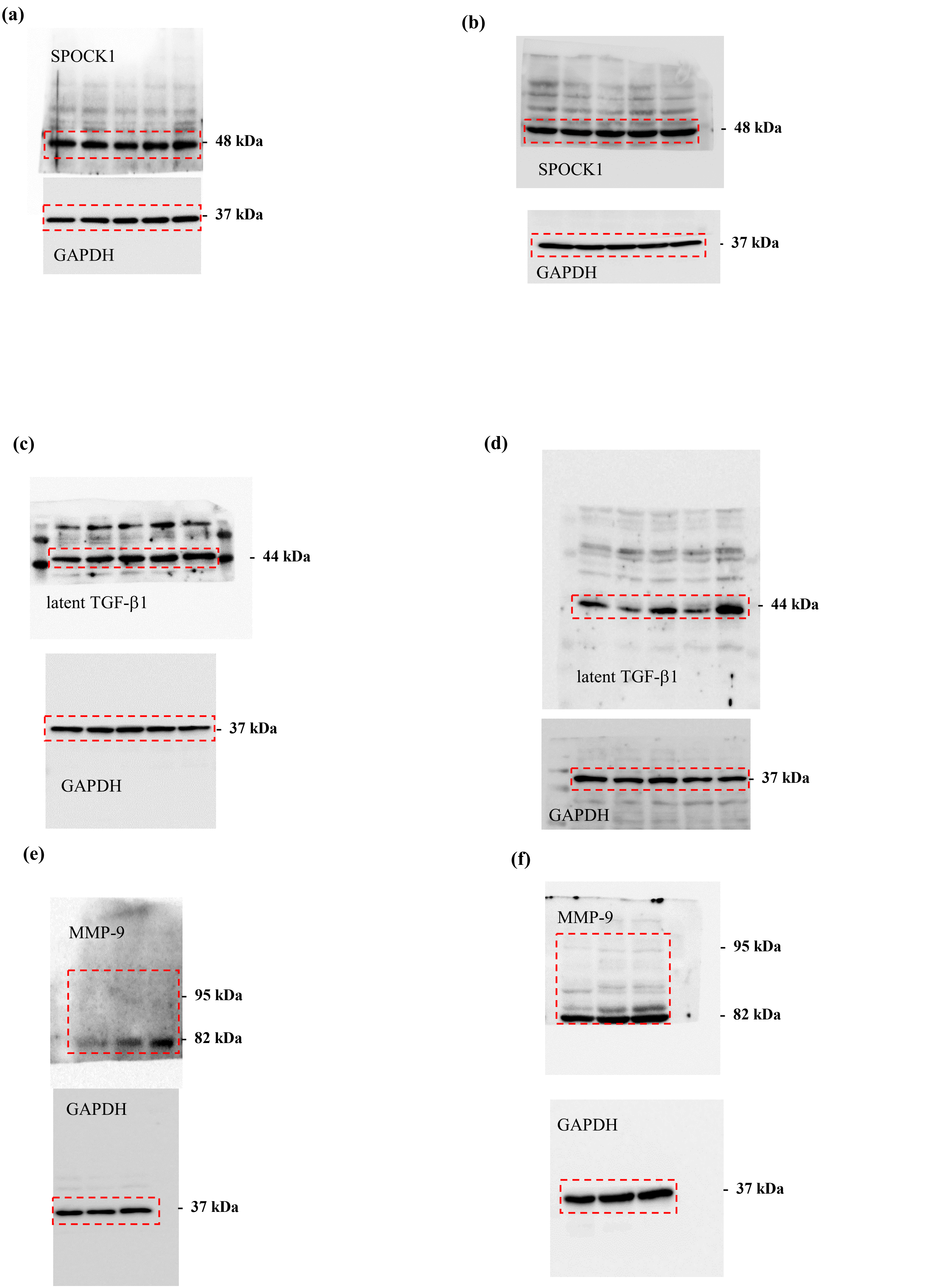
(a)
SPOCK1
48 kDa
37 kDa
GAPDH
(b)
48 kDa
SPOCK1
37 kDa
GAPDH
(c)
44 kDa
latent TGF-β1
37 kDa
GAPDH
(d)
44 kDa
latent TGF-β1
37 kDa
GAPDH
(e)
MMP-9
95 kDa
82 kDa
GAPDH
37 kDa
(f)
MMP-9
95 kDa
82 kDa
GAPDH
37 kDa

Figure 5

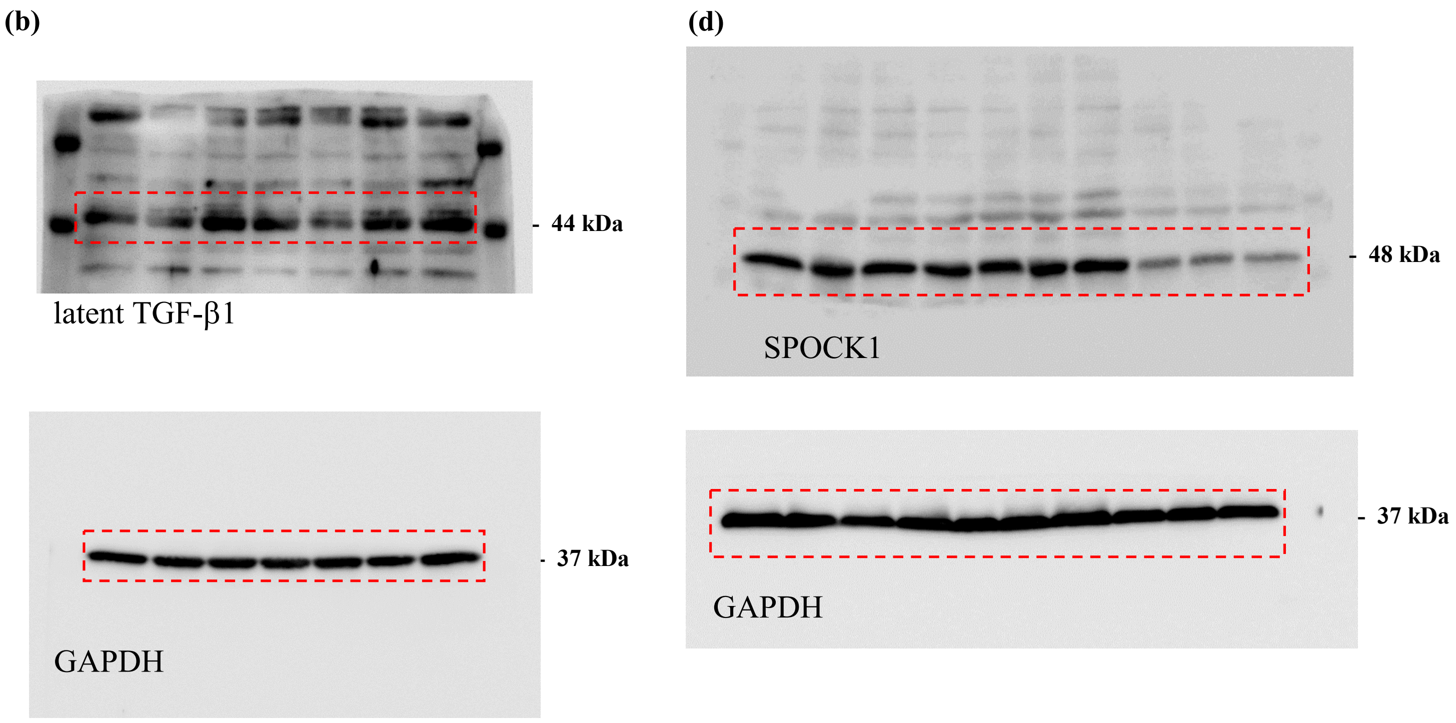

Figure 6

(a)

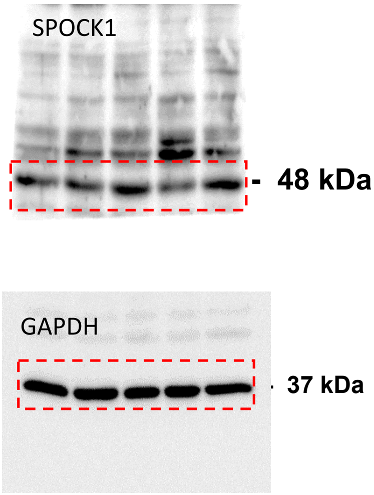

(b)

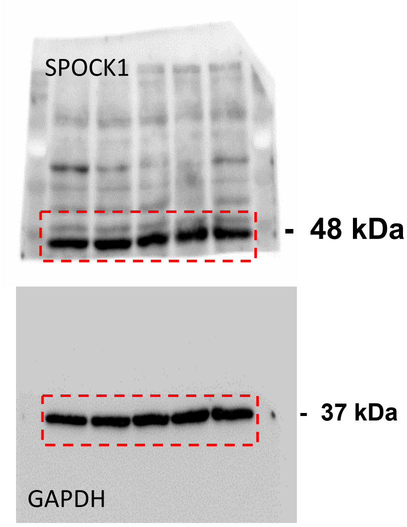

Supplementary Figure S1

(c)

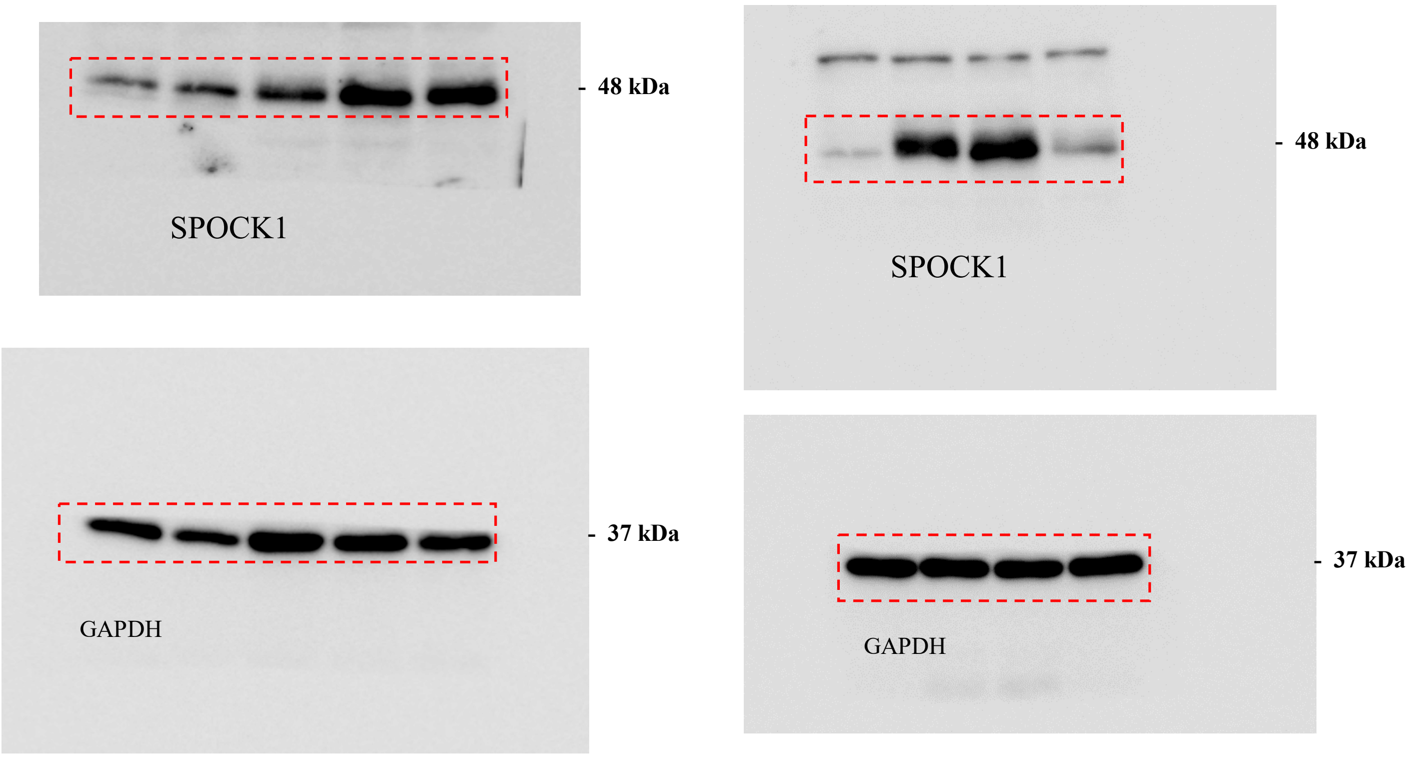

Supplement: Supplementary file 1 — Supplementary information [file 41598_2020_66660_MOESM1_ESM.pdf]
